# Spatially localized immune metaprograms reveal micro-niche organization in the human Dorsal Root Ganglion

**DOI:** 10.1371/journal.pone.0354750

**Published:** 2026-08-03

**Authors:** Kyuhyung Choi, Kaehong Lee, Baeki E. Kang, Tae-Min Kim

**Affiliations:** 1 Department of Medical Informatics, College of Medicine, The Catholic University of Korea, Seoul, Korea; 2 Bundang New York Animal Hospital, Seongnam, Republic of Korea; 3 Department of Anesthesiology and Pain Medicine, Seoul National University Hospital, Seoul, Korea; 4 CMC Institute for Basic Medical Science, The Catholic Medical Center of The Catholic University of Korea, Seoul, Republic of Korea; 5 Cancer Research Institute, College of Medicine, The Catholic University of Korea, 222 Banpo-daero, Seocho-Gu, Seoul, Korea; PLOS ONE, UNITED KINGDOM OF GREAT BRITAIN AND NORTHERN IRELAND

## Abstract

Dorsal root ganglion (DRG)–targeted local interventions are widely used in pain management; however, the cellular and spatial organization of immune transcriptional states within human DRG tissue remains poorly defined. In particular, it is unclear whether DRG immune activity reflects a single dominant inflammatory axis or a spatially heterogeneous microenvironment composed of parallel transcriptional programs. To address this question, we analyzed publicly available human DRG single-nucleus RNA sequencing data (GSE189501) to identify immune transcriptional metaprograms using rank-stable non-negative matrix factorization (NMF). A curated subset of 388 immune nuclei was analyzed to minimize lineage contamination, and rank selection was evaluated across k = 4–9, with k = 7 selected based on consensus stability metrics. Metaprograms were characterized using gene loading patterns, differential expression between high- and low-scoring cells, and Hallmark gene set enrichment analysis. Identified gene sets were then projected onto human DRG Xenium spatial transcriptomics data (GSE273557) by recalculating program scores from spatial transcript counts, and spatial aggregation of program-high cells was quantified using nearest-neighbor distance analysis with permutation-based significance testing. To evaluate robustness, the program gene signatures were further projected onto an independent human DRG snRNA-seq dataset (GSE168243). Unsupervised NMF identified seven reproducible immune metaprograms that did not converge on a single dominant inflammatory signature but instead represented parallel transcriptional states with distinct gene-loading patterns and functional annotations. One program exhibited receptor- and sensing-associated transcriptional features without strong classical inflammatory enrichment. Spatial projection revealed region-specific enrichment of metaprogram-high cells, and nearest-neighbor analysis demonstrated significant spatial aggregation in selected regions, supporting localized immune micro-niche organization rather than uniform tissue-wide activation. These findings indicate that human DRG immune organization is composed of multiple parallel transcriptional programs with spatially localized enrichment patterns, supporting a model of micro-niche–based immune architecture that may provide biological context for DRG-targeted local interventions.

## 1. Introduction

The dorsal root ganglion (DRG) occupies a central position in peripheral sensory processing by housing the cell bodies of primary afferent neurons [1]. Beyond its neuronal function, the DRG represents a structurally unique neuroimmune interface [2], where neuronal, glial, vascular, and immune components coexist in close anatomical proximity [3]. This distinctive organization places the DRG at the intersection of sensory transmission and local immune regulation [1].

In clinical practice, the DRG is frequently targeted through local interventions [4], including selective nerve root blocks, epidural injections, and neuromodulation strategies [5–7]. Corticosteroids such as dexamethasone [8] are widely administered based on their broad anti-inflammatory effects. However, the biological organization of immune transcriptional states within the human DRG remains incompletely defined [9]. As a result, the mechanistic basis for DRG-targeted interventions is often extrapolated from central nervous system paradigms [10] rather than grounded in DRG-specific tissue biology.

Mechanistic studies of chronic pain have historically emphasized spinal cord–centric models, particularly microglial activation and Apoe-associated immune transcriptional states [11]. These central immune reprogramming frameworks have significantly advanced the understanding of neuropathic pain [12]. Nevertheless, their direct applicability to the peripheral nervous system, and to the DRG specifically, remains uncertain.

The DRG differs fundamentally from the spinal cord in its immune composition and tissue architecture [13]. Microglia, the resident immune cells of the central nervous system, are absent in the DRG. Instead, the DRG contains macrophages, satellite glial cells, Schwann cells, endothelial cells, and stromal components within a relatively permeable vascular environment [14]. These structural and immunological distinctions suggest that immune organization in the DRG may not conform to a single dominant inflammatory axis analogous to that described in spinal cord tissue [15–17].

Recent advances in single-nucleus RNA sequencing (snRNA-seq) [18] and spatial transcriptomics [19] provide new opportunities to examine immune organization within intact human DRG tissue. While single-cell approaches enable unsupervised identification of transcriptional programs, they do not preserve anatomical context [20]. Conversely, spatial transcriptomics preserves tissue architecture but requires prior definition of biologically meaningful gene programs for projection-based analysis. Integrating these modalities allows characterization of both transcriptional heterogeneity and spatial patterning without presupposing discrete immune polarization states [21].

In this study, we define immune transcriptional metaprograms in human DRG using rank-stable non-negative matrix factorization and subsequently project these programs onto human DRG Xenium spatial transcriptomics data. Rather than seeking a dominant inflammatory signature, we evaluate whether immune transcriptional states organize into spatially localized micro-niches within DRG tissue. Through this integrative approach, we aim to provide a spatially grounded framework for understanding immune organization in the human DRG and to establish biological context for DRG-targeted local interventions beyond uniform anti-inflammatory models.

To evaluate the robustness of the inferred immune metaprograms, we additionally projected the program gene signatures onto an independent human DRG single-nucleus RNA sequencing dataset. This external validation was performed to assess whether the metaprogram architecture identified in the discovery dataset is reproducible across independent cohorts.

## 2. Methods

### 2.1. Study design and analytical workflow

This study was designed as a mechanistic transcriptomic investigation to characterize immune transcriptional programs in the human dorsal root ganglion (DRG) and to evaluate their spatial organization within intact tissue architecture. Rather than assessing therapeutic efficacy or clinical outcomes, the study aimed to determine whether DRG immune activity is organized as a single dominant inflammatory axis or as multiple parallel transcriptional programs exhibiting localized spatial enrichment. The analytical workflow consisted of three major steps. First, immune transcriptional metaprograms were identified from human DRG single-nucleus RNA sequencing (snRNA-seq) data (GSE189501) using rank-stable non-negative matrix factorization (NMF). Second, the resulting metaprograms were projected onto human DRG Xenium spatial transcriptomics data (GSE273557) to evaluate spatial localization and micro-niche organization. Third, the reproducibility of the inferred metaprogram architecture was assessed in an independent human DRG snRNA-seq dataset (GSE168243). All analyses were performed within human datasets, and no cross-species integration was conducted. This study exclusively used publicly available, de-identified datasets and did not involve new human subjects’ research. No identifiable personal information was accessed or analyzed. Accordingly, institutional review board approval and informed consent were not required for this secondary data analysis.

### 2.2. Computational environment

All computational analyses were performed locally using R version 4.4.3. Major packages included Seurat for preprocessing, normalization, clustering, dimensionality reduction, and module scoring; NMF for transcriptional program decomposition and rank stability assessment; ComplexHeatmap for visualization of gene loading patterns; fgsea and msigdbr for pathway enrichment analyses; Matrix for sparse matrix operations; arrow for handling Xenium parquet files; and spatstat.geom and FNN for spatial coordinate processing and nearest-neighbor analyses. Data manipulation and figure generation were performed using tidyverse packages including dplyr and ggplot2. Exact package versions are available through the sessionInfo() function.

### 2.3. Public datasets

Human DRG single-nucleus RNA sequencing data were obtained from the Gene Expression Omnibus (GEO) under accession GSE189501. This dataset provided processed count matrices and cell-level metadata used for immune metaprogram discovery. Human DRG Xenium spatial transcriptomics data were obtained from GSE273557 and included cell-level centroid coordinates, transcript-level detections, segmentation information, and associated histological images. Spatial analyses were restricted to DRG tissue regions provided in the original dataset. To evaluate reproducibility, an independent human DRG snRNA-seq dataset (GSE168243) was used as an external validation cohort. Discovery, spatial projection, and validation analyses were performed independently for each dataset.

### 2.4. snRNA-seq preprocessing and definition of TRUE immune nuclei

The GSE189501 count matrix was processed using Seurat. Standard quality-control metrics including the number of detected genes per nucleus (nFeature_RNA), total transcript counts (nCount_RNA), and mitochondrial transcript percentage (percent.mt) were calculated. Cells failing predefined quality-control thresholds were removed prior to downstream analyses. Data were normalized using NormalizeData, and highly variable genes were identified using FindVariableFeatures with the variance-stabilizing transformation method. Scaled expression matrices were generated using ScaleData, followed by principal component analysis (PCA), graph-based clustering, and UMAP visualization.

To minimize contamination from non-immune populations, a curated set of TRUE immune nuclei was defined prior to NMF analysis. Candidate immune cells were initially identified using lineage-specific marker expression, after which nuclei exhibiting residual neuronal signatures were excluded. A fixed list of 388 curated immune nuclei was retained, and only these nuclei were used for subsequent metaprogram discovery. This filtering strategy was implemented to ensure that identified transcriptional programs reflected immune-specific biological variation rather than residual signals from neighboring neuronal populations.

### 2.5. Identification of immune metaprograms

#### 2.5.1. Rationale for NMF-based program discovery.

Non-negative matrix factorization (NMF) was applied to the curated TRUE immune population not to redefine canonical immune cell types, but to identify transcriptional programs that could be projected onto spatial transcriptomic data. The Xenium panel used in this study contained a restricted feature space (panel97), derived from the intersection between the Xenium 100-gene panel and the snRNA-seq dataset. Because this constrained gene set lacked many canonical subtype-defining immune markers, fine-grained immune subtype annotation was inherently limited. Under these conditions, program-based decomposition provides a more robust representation of coordinated transcriptional activity than subtype classification. Accordingly, NMF-derived programs were interpreted as orthogonal transcriptional axes representing co-expressed biological states rather than surrogate immune subtypes.

#### 2.5.2. NMF implementation and rank selection.

The NMF input matrix was constructed from log-normalized expression values obtained from the RNA assay data layer. Only genes present within the predefined panel97 feature space were retained. Negative values were truncated to zero and a small constant (1 × 10^-9) was added to ensure numerical stability. NMF was performed using the Brunet algorithm implemented in the NMF package with 30 independent runs per rank (nrun = 30). Parallel execution was disabled to ensure deterministic behavior.

Candidate ranks ranging from k = 4 to k = 9 were evaluated using nmfEstimateRank. Rank selection was based on multiple stability metrics including cophenetic correlation coefficient, dispersion, silhouette index, and consensus matrix structure. Consensus matrices were reordered using hierarchical clustering based on one minus consensus distance. Based on the combined stability and separation characteristics, k = 7 was selected as the optimal rank for immune metaprogram identification.

#### 2.5.3. Characterization of immune metaprograms.

The NMF basis matrix (W; genes × programs) was used to identify program-defining genes. For each metaprogram, genes were ranked according to loading weight, and the top-loading genes were used for downstream interpretation. Gene loading heatmaps were generated using row-wise z-score scaling.

Cell-level program activity was quantified using the NMF coefficient matrix (H; programs × cells). Coefficients were column-normalized to obtain relative program contributions within individual cells. The dominant program for each cell was defined as the program with the highest normalized coefficient. For analyses focusing on IMM7_P4, cells within the highest decile of IMM7_P4 scores were designated as P4-high cells.

Differential expression between P4-high and P4-low populations was performed using Seurat FindMarkers with Wilcoxon rank-sum testing. Only genes detected in at least 5% of cells were considered. Ranked gene lists based on log2 fold-change values were subjected to Hallmark pathway enrichment analysis using fgseaMultilevel. Hallmark gene sets were obtained from MSigDB through the msigdbr package. Enrichment results were summarized using normalized enrichment scores and Benjamini–Hochberg adjusted p-values.

### 2.6. Spatial projection of immune metaprograms

#### 2.6.1. Program score recalculation in Xenium spatial transcriptomics data.

For each Xenium region (reg1–reg4), cell-level expression matrices were extracted from the Xenium assay. Metaprogram scores were recalculated directly from spatial transcript counts using the top genes identified from the corresponding NMF metaprogram. For each cell, the metaprogram score was defined as the mean value of log(1 + counts) across all genes belonging to the corresponding signature. Score calculations were performed using segmentation-level cell identifiers (cell_id) to maintain compatibility with Xenium Explorer.

#### 2.6.2. Xenium Explorer visualization.

Two classes of Xenium Explorer-compatible CSV files were generated for each region. Continuous score files contained cell-level metaprogram scores (IMM7_P1–IMM7_P7) and were used for gradient-based visualization. Binary classification files were generated by ranking cells according to metaprogram score and assigning the top 10% of cells to a program-high group while all remaining cells were assigned to a program-other group. For IMM7_P4, group labels were recorded as IMM7_P4_top10_high and IMM7_P4_top10_other. These files were imported into Xenium Explorer using the Custom Cell Groups functionality.

#### 2.6.3. Spatial clustering analysis.

To quantify spatial aggregation of metaprogram-high cells, nearest-neighbor distance analysis was performed using cell centroid coordinates obtained from Xenium segmentation outputs. For each region, the mean nearest-neighbor distance among program-high cells was calculated. A null distribution was generated by randomly sampling the same number of cells from all segmented cells within the corresponding tissue region. This procedure was repeated 1,000 times for each analysis.

Spatial clustering was summarized using a clustering ratio defined as the observed mean nearest-neighbor distance divided by the mean nearest-neighbor distance obtained from the null distribution. Values below one indicate increased spatial aggregation relative to random expectation. Permutation-based p-values were calculated as the proportion of random samples with mean nearest-neighbor distances less than or equal to the observed value.

#### 2.6.4. Projection robustness analysis.

To evaluate the robustness of metaprogram projection within the constrained panel97 feature space, sensitivity analyses were performed for the IMM7_P4 signature. First, leave-one-gene-out analysis was conducted by sequentially removing each signature gene and recalculating program scores. Correlations between perturbed and original scores were quantified using Pearson and Spearman coefficients. Second, random gene dropout analysis was performed by removing 10% of IMM7_P4 genes across 1,000 independent iterations. Program scores were recalculated after each perturbation and compared with the original scores. Stability of P4-high classification was further evaluated by quantifying the overlap and Jaccard similarity between the original and perturbed top-decile cell assignments.

### 2.7. External validation of immune metaprograms

To evaluate the reproducibility of the inferred immune metaprograms, an independent human DRG single-nucleus RNA sequencing dataset (GSE168243) was analyzed. Metaprogram gene signatures derived from the discovery dataset were projected onto the validation dataset using module score-based gene set projection. For each IMM7 program, the corresponding signature genes were used to calculate program scores across validation samples. Program-level activity patterns and dominant program distributions were then compared between discovery and validation cohorts to assess preservation of the overall metaprogram architecture.

### 2.8. Statistical analysis and interpretation

All statistical analyses were performed in R version 4.4.3. Differential expression analyses were conducted using Wilcoxon rank-sum testing. Pathway enrichment significance was assessed using fgseaMultilevel and adjusted using the Benjamini–Hochberg procedure. Spatial clustering significance was evaluated using permutation-based testing. Unless otherwise stated, p-values less than 0.05 were considered statistically significant.

Metaprograms were interpreted as transcriptional states derived from unsupervised decomposition rather than predefined immune polarization categories. Similarly, spatial clustering results were interpreted conservatively as evidence of localized enrichment and micro-niche organization, without inferring causal signaling relationships or hierarchical immune state transitions.

## 3. Results

### 3.1. Overview of analytical outputs

**Fig 1:**
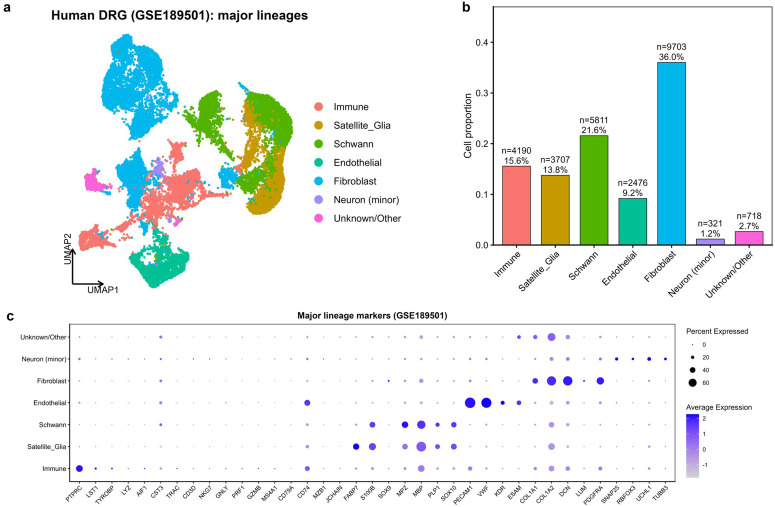
Global cellular landscape of human DRG revealed by single-nucleus RNA sequencing. **(A)** Uniform manifold approximation and projection (UMAP) of human DRG single-nucleus RNA sequencing data (GSE189501) revealed a reproducible multi-lineage cellular architecture. **(B)** Major cell classes included neurons, satellite glial cells, Schwann cells, endothelial cells, stromal populations, and immune nuclei. **(C)** Marker-based annotation confirmed lineage-specific transcriptional identities. Neuronal nuclei were transcriptionally distinct from immune and glial populations, enabling focused downstream analysis of immune heterogeneity. Immune nuclei formed a coherent but internally heterogeneous cluster, providing the foundation for transcriptional decomposition using NMF.

We first defined the global cellular landscape of the human dorsal root ganglion (DRG) using single-nucleus RNA sequencing (Fig 1). Immune nuclei were then isolated and subjected to unsupervised transcriptional decomposition. The resulting immune metaprograms were functionally characterized at the gene level. Finally, selected metaprograms were projected onto spatial transcriptomics data to evaluate their anatomical distribution and to quantify spatial aggregation.

#### 3.1.1. Immune nuclei constitute a transcriptionally distinct yet heterogeneous compartment.

Single-nucleus RNA sequencing of human DRG tissue resolved major cellular lineages, including neuronal, glial, vascular, stromal, and immune populations. Immune nuclei formed a transcriptionally distinct compartment separate from neuronal and glial lineages, allowing immune-focused analyses without cross-lineage interference.

Within this immune compartment, transcriptional heterogeneity was evident, suggesting the presence of multiple underlying states rather than a uniform immune profile. This observation motivated unsupervised decomposition to resolve structured transcriptional programs.

### 3.2. Rank-stable decomposition identifies seven immune metaprograms

**Fig 2:**
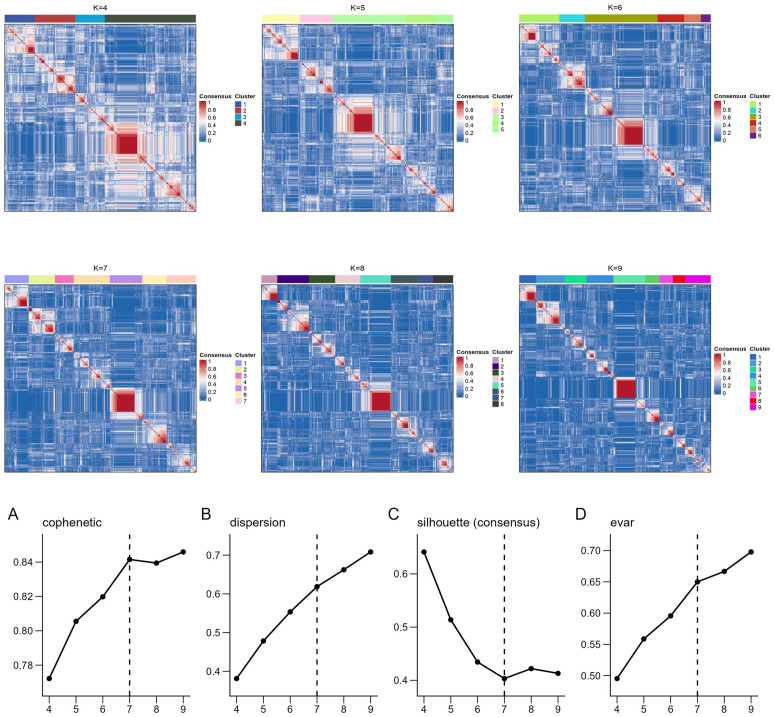
Determination of optimal rank for immune metaprogram decomposition. Non-negative matrix factorization was applied to a curated subset of 388 TRUE immune nuclei. Rank selection was evaluated across k values from 4 to 9 using stability metrics, including cophenetic correlation, dispersion, and consensus matrix structure (lower panel A, B, C, D). Stability improved from k = 4 to k = 7 and declined beyond k = 7, where fragmentation and reduced reproducibility were observed. Consensus matrix visualization demonstrated clear block-diagonal structure at k = 7, indicating robust separation of transcriptional states. Based on these criteria, k = 7 was selected as the optimal rank for immune metaprogram identification.

Application of non-negative matrix factorization to 388 curated immune nuclei revealed that transcriptional structure was optimally resolved at k = 7. Stability metrics supported this rank as providing reproducible partitioning without over-fragmentation (Fig 2).

**Fig 3:**
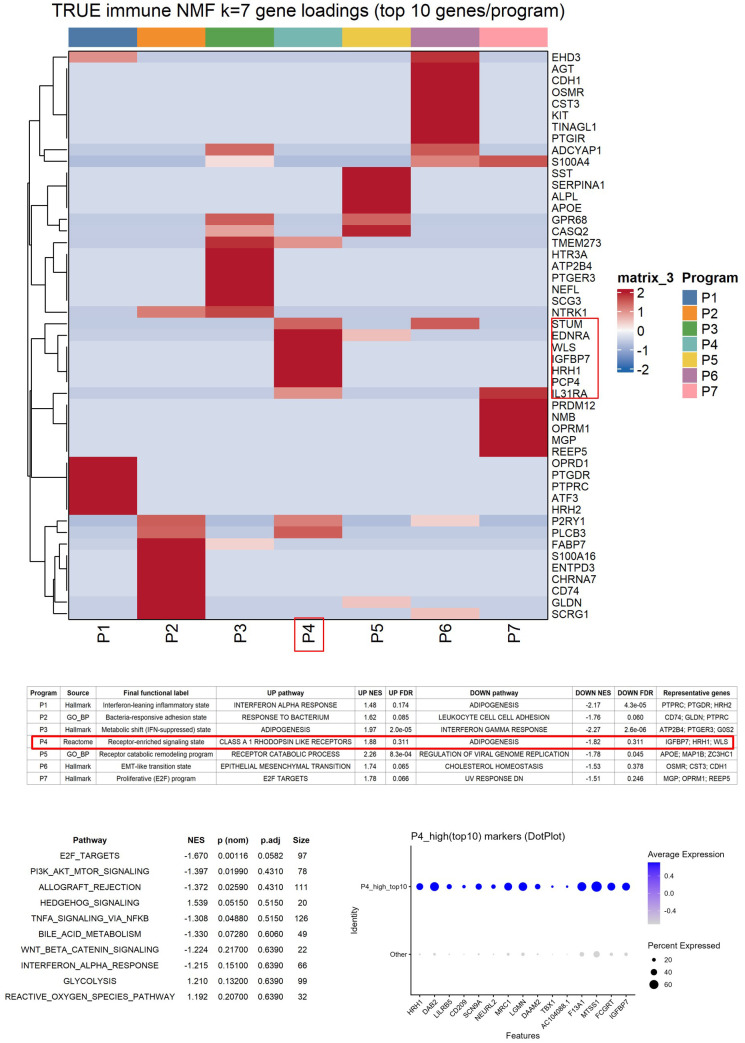
Gene-level structure and functional annotation of immune metaprograms. The k = 7 NMF solution identified seven immune transcriptional metaprograms (IMM7_P1 through IMM7_P7), each characterized by distinct gene loading patterns. Heatmap visualization of top-loading genes demonstrated clear separation among programs (upper panel). Cell-level program scoring revealed that immune nuclei were distributed across multiple transcriptional states rather than converging into a single dominant inflammatory program. Differential expression analysis between high- and low-scoring cells confirmed coherent gene-level differences within each program (middle panel). Functional enrichment analysis indicated heterogeneous pathway associations across metaprograms. Notably, IMM7_P4 exhibited receptor- and sensing-associated transcriptional features without strong enrichment for canonical pro-inflammatory Hallmark pathways. Collectively, these findings indicate that immune organization within the human DRG is structured as parallel transcriptional programs rather than a unified inflammatory axis (lower panel).

The resulting seven metaprograms represented separable transcriptional patterns distributed across immune nuclei. Importantly, cell-level program assignment demonstrated that immune cells were partitioned across multiple states rather than converging into a single dominant transcriptional program (Fig 3).

### 3.3. Parallel transcriptional programs without emergence of a dominant inflammatory axis

Gene-level analyses confirmed that each metaprogram captured coherent transcriptional structure. Differential expression between high- and low-scoring cells within individual programs identified consistent gene-level shifts, indicating biologically meaningful separation.

Functional enrichment analyses demonstrated heterogeneous pathway associations across metaprograms. Although immune-related pathways were represented among specific programs, no single program exhibited characteristics of global inflammatory dominance across the immune compartment.

IMM7_P4 was enriched for genes associated with histaminergic (HRH1, HRH2), purinergic (P2RY1), neurotrophic (RET, GFRA2), growth factor–related (IGFBP7), and sensory signaling pathways (SCN9A, CALCA), suggesting a receptor-mediated environmental sensing and neuroimmune communication state rather than a classical inflammatory program. This pattern supports a model in which immune transcriptional diversity in the DRG is structured as parallel programs rather than as a unified inflammatory axis [22].

### 3.4. Spatial projection reveals non-random micro-niche organization

**Fig 4:**
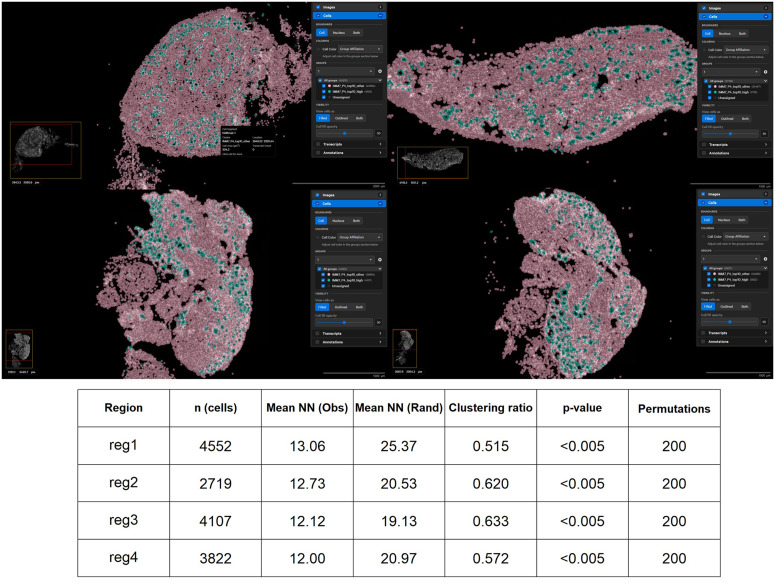
Spatial projection and micro-niche clustering of IMM7_P4 in human DRG. To examine anatomical organization, the IMM7_P4 gene set was projected onto human DRG Xenium spatial transcriptomics data (GSE273557). Program scores were recalculated directly from spatial counts, and the top 10 percent of cells by IMM7_P4 score were designated as P4-high. Visualization in Xenium Explorer demonstrated that P4-high cells were not uniformly distributed throughout the ganglion. Instead, localized regions of enrichment were observed within the broader DRG architecture (upper panel). To formally evaluate spatial aggregation, nearest-neighbor distance analysis with permutation testing was performed. The observed mean nearest-neighbor distance among P4-high cells was compared to null distributions generated by random sampling. In selected regions, P4-high cells exhibited significantly reduced nearest-neighbor distances relative to random expectation, yielding clustering ratios below 1 and statistically significant permutation-based p-values (lower panel). These results support the presence of localized immune micro-niches defined by specific transcriptional programs within the human DRG.

To determine whether immune transcriptional programs exhibit spatial structure, the IMM7_P4 gene set was projected onto human DRG Xenium spatial transcriptomics data. Cells with high IMM7_P4 scores were not uniformly distributed across tissue sections. Instead, region-specific enrichment patterns were observed (Fig 4).

Quantitative nearest-neighbor permutation testing confirmed that, in selected regions, IMM7_P4-high cells were positioned closer to one another than expected under random spatial assignment. Reduced mean nearest-neighbor distances relative to null distributions indicated statistically significant spatial aggregation.

These findings demonstrate that immune transcriptional heterogeneity in the human DRG is spatially organized rather than diffusely distributed, consistent with localized micro-niche architecture.

### 3.5. Independent validation of immune metaprograms in an external DRG dataset

To assess whether the transcriptional programs identified in the discovery dataset were reproducible, we projected the IMM7 program gene signatures onto an independent human DRG snRNA-seq dataset (GSE168243). Despite differences in experimental preparation and sequencing batches, the projected program scores revealed a comparable metaprogram architecture across samples (S1 Table). In particular, multiple programs, including the receptor/sensing-associated IMM7_P4 program, remained detectable across the independent dataset.

Consistent with the discovery cohort, the external dataset displayed heterogeneous combinations of dominant programs rather than a single linear inflammatory trajectory (S1 Fig). These results support the robustness of the inferred immune metaprogram structure and suggest that the parallel immune states observed in the discovery dataset are not dataset-specific artifacts.

## 4. Discussion

The present study situates immune organization in the human dorsal root ganglion within a broader neuroimmune context that has historically been shaped by central nervous system–centric paradigms [23,24]. In spinal cord models of neuropathic pain, immune activation is often described as converging toward dominant transcriptional states, frequently associated with microglial reprogramming. Such frameworks have been highly influential in conceptualizing pain as a centrally sustained inflammatory process.

However, the DRG occupies a distinct anatomical and immunological niche [25]. It lacks microglia and instead contains macrophages and other myeloid populations embedded within a ganglionic structure that is neither fully peripheral nor fully central [26]. The transcriptional architecture identified here suggests that immune heterogeneity in the DRG is not organized around a single axis of dominance but instead reflects coexisting and partially overlapping transcriptional programs. This distributed organization challenges the assumption that chronic pain–related immune processes must manifest as uniform inflammatory expansion.

Rather than representing a peripheral analogue of spinal microglial activation, DRG immune organization may be better understood as a structured ensemble of parallel states that remain spatially and transcriptionally differentiated [27,28].

A central conceptual contribution of this work is the integration of transcriptional decomposition with spatial positioning. Immune heterogeneity is commonly interpreted in terms of differential gene expression profiles alone. By embedding transcriptional programs within intact ganglionic architecture, the present analysis emphasizes that spatial context provides an additional axis of biological organization.

The observation that selected transcriptional programs exhibit localized aggregation suggests that immune states in the DRG may be organized within spatially constrained micro-environments [29]. Such micro-niches may arise from local neuron–immune interactions, vascular gradients, or stromal organization. Importantly, spatial aggregation does not imply functional dominance; rather, it indicates non-random positioning within tissue architecture.

This perspective reframes immune heterogeneity as a spatially patterned phenomenon rather than a purely transcriptomic classification. In doing so, it aligns DRG biology with broader concepts of tissue micro-environments observed in other organ systems.

Another implication of these findings is the absence of clear polarization into a single pro-inflammatory state [30,31]. In many neuroimmune models, disease progression is conceptualized as a shift toward a dominant immune phenotype. The parallel transcriptional architecture observed here suggests an alternative framework: immune states may coexist without collapsing into a single hierarchical endpoint.

This form of functional parallelism is compatible with dynamic and context-dependent immune modulation [32]. It allows for localized immune engagement without necessitating ganglion-wide inflammatory transformation. Conceptually, this distributed model may better reflect the anatomical constraints and mixed cellular composition of the DRG.

Importantly, this interpretation remains conservative. The study does not assign causality to specific transcriptional programs, nor does it define stable immune subtypes. Instead, it proposes that immune organization in the DRG may be structured through spatially differentiated yet coexisting programs [33].

The application of NMF to immune nuclei warrants clarification, as such approaches are often used to further subdivide malignant or otherwise homogeneous cellular populations [34]. In the present study, NMF was not intended to redefine canonical immune subtypes. Rather, it was employed to extract structured transcriptional programs within a constrained gene panel space derived from the Xenium 100-gene platform. Because panel97 contains limited canonical subtype-defining markers, fine-grained immune subtype annotation is intrinsically restricted (S2 Fig). Under these resolution constraints, clustering-based lineage refinement becomes unreliable. Program-based decomposition instead provides a stable, orthogonal representation of co-expressed inflammatory modules that can be quantitatively projected onto spatial coordinates [35]. Importantly, the identified metaprograms did not collapse into surrogate lineage identities, but represented parallel transcriptional axes, supporting their interpretation as functional states rather than refined cell-type categories.

Importantly, the high proportion of unassigned cells reflects limited subtype-marker coverage within the constrained panel97 feature space rather than evidence of biologically undefined immune populations. The observation that multiple broad immune lineages contribute to individual metaprograms supports interpretation of these programs as transcriptional states rather than unresolved cell-type clusters.

The structured but non-dominant immune organization described here provides a biological context for DRG-targeted local interventions. If the DRG were uniformly inflamed, one might expect global inflammatory signatures to dominate the immune landscape. The absence of such dominance suggests that therapeutic interactions are likely to occur within a heterogeneous and spatially organized microenvironment [26,29].

This does not imply that specific agents preferentially target particular transcriptional programs, nor does it establish therapeutic efficacy. Rather, it supports a shift in conceptual framing: the DRG should not be viewed as a homogeneously inflamed tissue analogous to certain central models, but as a spatially structured neuroimmune interface.

Within such an interface, local interventions may encounter regionally enriched transcriptional environments. Whether and how these environments influence pain phenotypes remains an open question. Although IMM7_P4 exhibited enrichment of receptor- and sensing-associated genes, the present study does not establish direct neuron–immune communication or causal involvement in pain signaling. Rather, these findings identify a transcriptional state that may provide a framework for future mechanistic investigation of neuroimmune interactions within the human DRG. Although donor-level generalization cannot be established from the currently available dataset, the consistent detection of IMM7_P4 spatial aggregation across four independent Xenium regions suggests that the observed micro-niche organization is not restricted to a single tissue section or isolated spatial observation.

The analytical framework employed here emphasizes structural inference rather than mechanistic attribution. Unsupervised decomposition identifies reproducible transcriptional programs, but does not determine lineage commitment or functional causality. Spatial clustering statistics quantify non-random organization, yet do not specify the biological drivers of aggregation [36].

The integration of transcriptomic and spatial modalities provides a quantitative scaffold for interpreting DRG immune organization. Nevertheless, interpretation must remain bounded by the observational nature of the data and by the technical constraints of current spatial platforms.

Importantly, projection of the program gene signatures onto an independent human DRG snRNA-seq dataset reproduced a comparable metaprogram architecture, indicating that the identified immune metaprogram architecture is reproducible across independent cohorts and therefore unlikely to represent dataset-specific artifacts.

An important limitation of the present study is that the identified micro-niches are defined operationally through localized enrichment of transcriptional programs rather than through direct characterization of their cellular composition. Consequently, the current analyses do not determine whether these regions are preferentially associated with satellite glial cells, specific neuronal subtypes, vascular structures, or other anatomical landmarks. Future studies incorporating higher-dimensional spatial profiling and expanded marker coverage will be necessary to resolve the cellular architecture of these transcriptional micro-niches.

## 5. Limitations

Several limitations should be acknowledged.

First, the study is based on publicly available single-nucleus RNA sequencing and spatial transcriptomics datasets generated through independent experimental workflows. Platform-specific differences in sequencing depth, transcript capture efficiency, and preprocessing pipelines may influence quantitative estimates of gene expression and metaprogram scores.

Second, immune metaprograms were defined using non-negative matrix factorization within a tested rank range. Although k = 7 demonstrated stable partitioning under multiple metrics, alternative rank selections could yield partially overlapping transcriptional structures. Accordingly, the identified metaprograms should be interpreted as stable representations within this analytical framework rather than definitive biological categories.

Third, spatial projection relied on a targeted Xenium gene panel. Because only a predefined set of genes is measured, spatial representation of metaprograms is necessarily incomplete relative to full transcriptomic resolution.

Fourth, spatial clustering analyses were performed on a limited number of tissue regions and do not capture inter-individual variability. The generalizability of micro-niche patterns across donors remains to be established.

Furthermore, the available datasets contained limited clinical metadata and were not designed to evaluate associations with chronic pain status, age-related changes, or sex-specific immune organization. Consequently, the identified metaprograms should be interpreted as a description of baseline immune architecture rather than as disease- or demographic-specific signatures. Future studies incorporating clinically annotated DRG cohorts will be required to determine whether these transcriptional programs vary according to pain phenotype, age, sex, or treatment history.

Finally, spatial aggregation of metaprogram-high cells does not imply functional dominance, mechanistic causality, or therapeutic relevance. The statistical framework quantifies non-random spatial organization but does not identify the biological drivers of clustering.

## 6. Conclusions and future directions

In summary, this study demonstrates that immune organization in the human dorsal root ganglion (DRG) is structured as multiple parallel transcriptional metaprograms rather than a single dominant inflammatory state. Using unsupervised transcriptomic decomposition and spatial projection, we identified reproducible immune programs that coexist within the DRG and exhibit localized spatial enrichment consistent with micro-niche organization.

Unlike previous DRG studies that primarily focused on cell-type cataloging or differential gene expression, the present work integrates transcriptional program discovery with spatial transcriptomic organization. Our findings indicate that immune heterogeneity in the DRG reflects not only transcriptional diversity but also spatial positioning within intact ganglionic architecture. These observations support a model in which the DRG functions as a spatially organized neuroimmune interface rather than a uniformly inflamed tissue compartment.

Although the present study does not establish causal mechanisms linking specific immune programs to pain phenotypes, it provides a biologically grounded framework for investigating neuroimmune interactions within the human DRG. The identification of spatially localized immune micro-niches may be particularly relevant for understanding how immune activity is organized within a clinically important target of pain-related interventions.

Future studies integrating higher-resolution spatial profiling, functional validation, and clinical correlation will be necessary to determine how these transcriptional micro-niches contribute to sensory processing, pain mechanisms, and treatment responses. Nevertheless, this work establishes a quantitative and spatially informed framework for studying immune organization in the human DRG and provides biological context for future mechanistic investigations and DRG-targeted therapeutic strategies.

## Supporting information

S1 Table Quantitative validation summary table. (PDF)

S1 Fig Independent validation of DRG immune metaprograms in an external dataset. (PDF)

S2 Fig Definition of TRUE immune cells and panel97-related limitations in fine immune subtype resolution. (PDF)
