# Supplementary material for: Spatially localized immune metaprograms reveal micro-niche organization in the human Dorsal Root Ganglion
Source: PLoS One. 2026 Aug 3;21(8):e0354750. doi: 10.1371/journal.pone.0354750 (PMC13432135; doi:10.1371/journal.pone.0354750)
Supplement: S1 Table — (PDF) [file pone.0354750.s001.pdf]

S1 Table. Quantitative validation summary table

Metric	Value
Validation cells	1496
Validation samples	6
Programs quantified	7
Mean genes used/program	26.8
Cell-level architecture correlation	0.855
Sample-level architecture correlation	0.902
Top-decile Jaccard	0.575

Quantitative validation summary of IMM7 metaprogram projection in GSE168243. On average, 26.8 of 30 signature genes per program were retained in the validation dataset. Program architecture was preserved at both the cell level (mean Spearman correlation = 0.855) and sample level (mean Spearman correlation = 0.902). Top-decile program assignment showed moderate concordance across programs (mean Jaccard index = 0.575), supporting reproducibility of the inferred metaprogram architecture.
