# Supplementary material for: Spatially localized immune metaprograms reveal micro-niche organization in the human Dorsal Root Ganglion
Source: PLoS One. 2026 Aug 3;21(8):e0354750. doi: 10.1371/journal.pone.0354750 (PMC13432135; doi:10.1371/journal.pone.0354750)
Supplement: S1 Fig — (PDF) [file pone.0354750.s002.pdf]

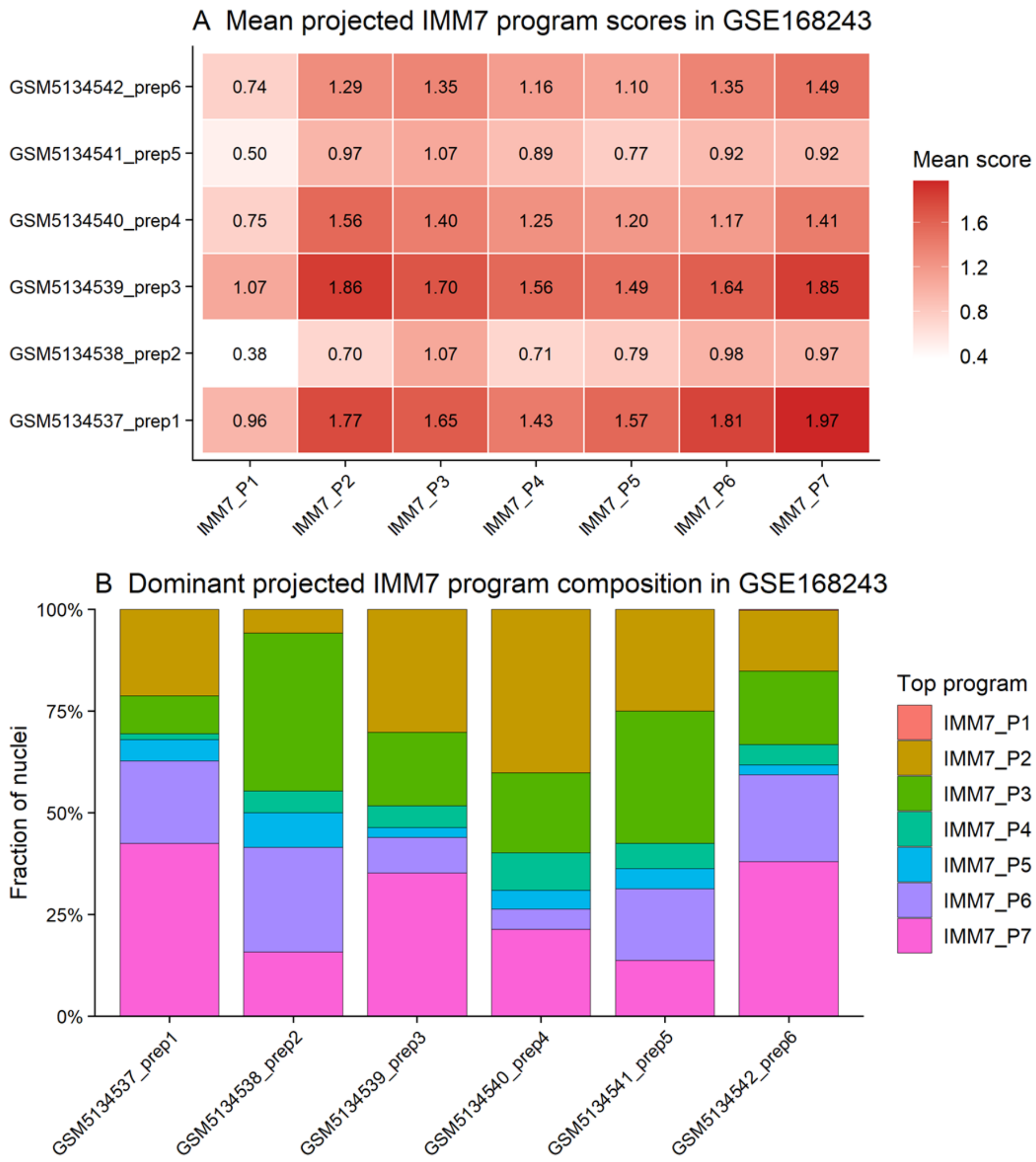

S1 Fig. Independent validation of DRG immune metaprograms in an external dataset.

(A) Projection of IMM7 program gene signatures onto an independent human DRG single-nucleus RNA-seq dataset (GSE168243). For each program, module scores were calculated using genes derived from the discovery dataset. Heatmap values represent the mean projected program scores across samples, demonstrating that multiple IMM7 programs are consistently detectable in the external dataset.

(B) Dominant projected IMM7 program composition across samples in the validation dataset. Each bar represents the fraction of nuclei assigned to each dominant IMM7 program based on the highest projected module score. Similar to the discovery dataset, the validation dataset displays heterogeneous combinations of programs rather than a single linear inflammatory trajectory, supporting the robustness of the inferred metaprogram architecture.
