# Supplementary material for: Spatially localized immune metaprograms reveal micro-niche organization in the human Dorsal Root Ganglion
Source: PLoS One. 2026 Aug 3;21(8):e0354750. doi: 10.1371/journal.pone.0354750 (PMC13432135; doi:10.1371/journal.pone.0354750)
Supplement: S2 Fig — (PDF) [file pone.0354750.s003.pdf]

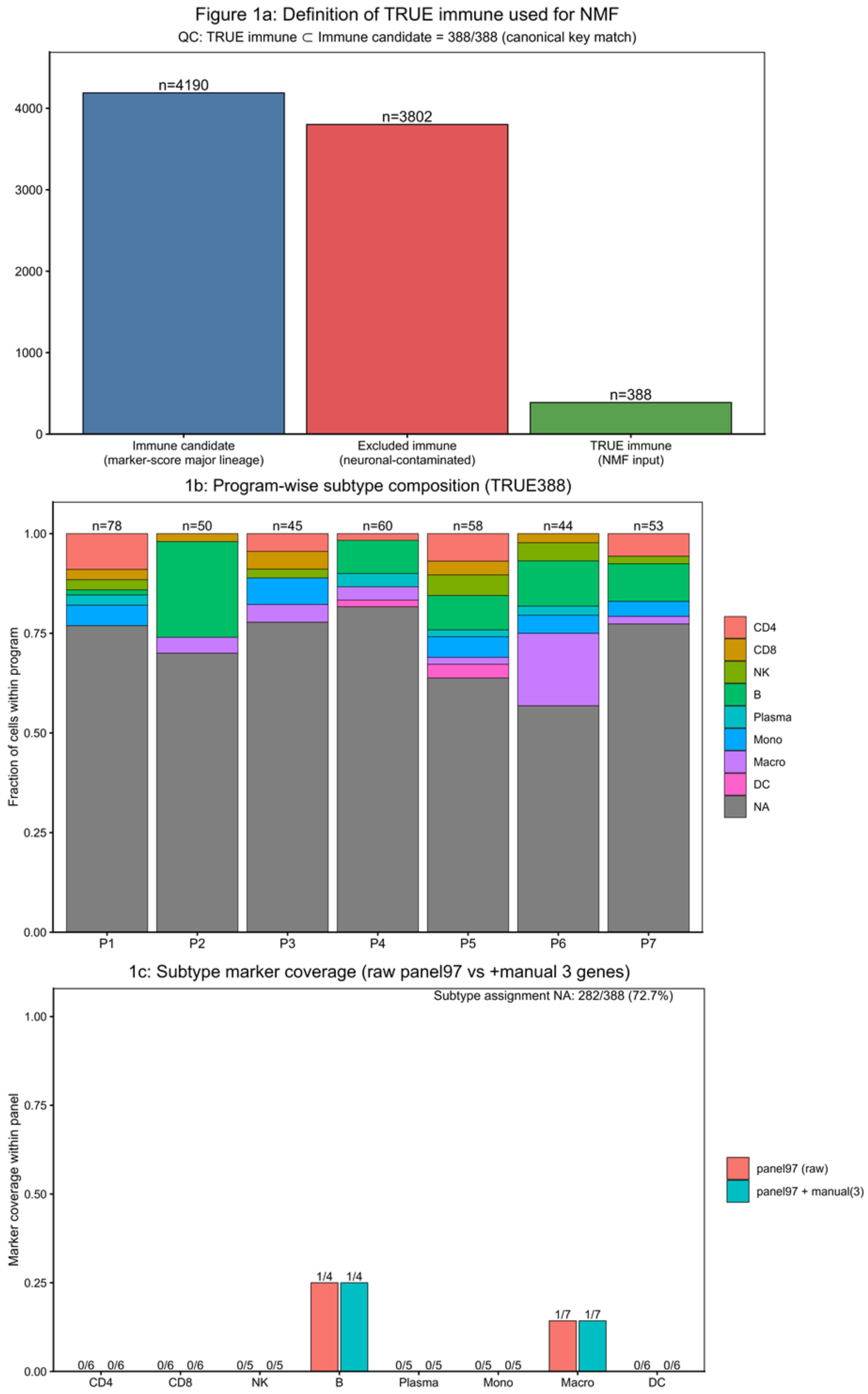

S2 Fig. Definition of TRUE immune cells and panel97-related limitations in fine immune subtype

resolution

(a) Immune candidate cells (n = 4,190) were defined from the GSE189501 snRNA-seq dataset using marker-score–based major lineage annotation. Immune cells exhibiting residual neuronal marker expression were considered neuron-contaminated and excluded (n = 3,802), yielding a curated set of TRUE immune cells (n = 388) used as input for NMF. TRUE immune cells were fully contained within the immune candidate population (388/388; canonical key match).

(b) For TRUE immune cells (n = 388), the dominant NMF program (P1–P7) was assigned per cell and program-wise subtype composition was visualized. Subtypes were defined using canonical immune marker genes and classified as CD4, CD8, NK, B, Plasma, Mono, Macro, DC, or NA (unassigned). The number of cells per dominant program is indicated above each bar.

(c) The Xenium custom 100-gene panel (GSE273557) was intersected with the snRNA feature set, resulting in a reduced 97-gene panel (panel97). Three genes—PLP1, FKBP5, and ANXA1—were absent from the intersected feature space (c1). Although these genes can be manually restored, subtype marker coverage within panel97 remained limited (c2), with most canonical subtype-defining genes absent from the restricted panel space. Consequently, fine immune subtype assignment exhibited a high proportion of unclassified cells (e.g., 282/388, 72.7%). These results indicate that the elevated NA rate primarily reflects insufficient marker coverage in the constrained panel97 gene space, rather than biological absence of defined immune subtypes.
