# Supplementary material for: Spatially localized immune metaprograms reveal micro-niche organization in the human Dorsal Root Ganglion
Source: PLoS One. 2026 Aug 3;21(8):e0354750. doi: 10.1371/journal.pone.0354750 (PMC13432135; doi:10.1371/journal.pone.0354750)
Supplement: Attachment — Submitted filename: Reviewer`s comments.docx [file pone.0354750.s004.docx]

1. **Introduction**

In the introduction, the biological interpretation provided is limited that is:

- Findings are described, but biological or clinical implications are not deeply explored.
- The “receptor- and sensing-associated program” is vague and not explained.
- Briefly explain:
  - What these programs represent biologically
  - Why spatial heterogeneity matters in DRG

2. **Results lack of quantitative highlights (statistical analysis)**

- No numbers summarizing key results (e.g., % variance explained, effect sizes, significance levels). Include 1–2 key metrics (e.g., significance, clustering strength, enrichment scores).

3. **Discussion**

In the discussion section make the impact explicit:

- How this informs pain mechanisms
- How it may influence targeted therapies

4. **Conclusion**

In the conclusion, please make the following revisions:

- Final sentence is descriptive rather than impactful.
- Does not clearly state *why this matters for patients or interventions*.
- Explicitly state what is new compared to prior DRG studies.
