# Supplementary material for: Spatially localized immune metaprograms reveal micro-niche organization in the human Dorsal Root Ganglion
Source: PLoS One. 2026 Aug 3;21(8):e0354750. doi: 10.1371/journal.pone.0354750 (PMC13432135; doi:10.1371/journal.pone.0354750)
Supplement: Attachment — Submitted filename: Response to Reviewers.docx [file pone.0354750.s006.docx]

Reviewers' comments:

Reviewer's Responses to Questions

Comments to the Author

1. Is the manuscript technically sound, and do the data support the conclusions?

The manuscript must describe a technically sound piece of scientific research with data that supports the conclusions. Experiments must have been conducted rigorously, with appropriate controls, replication, and sample sizes. The conclusions must be drawn appropriately based on the data presented.

Reviewer #1: Yes

Reviewer #2: Yes

2. Has the statistical analysis been performed appropriately and rigorously?

Reviewer #1: No

Reviewer #2: Yes

3. Have the authors made all data underlying the findings in their manuscript fully available?

The PLOS Data policy requires authors to make all data underlying the findings described in their manuscript fully available without restriction, with rare exception (please refer to the Data Availability Statement in the manuscript PDF file). The data should be provided as part of the manuscript or its supporting information, or deposited to a public repository. For example, in addition to summary statistics, the data points behind means, medians and variance measures should be available. If there are restrictions on publicly sharing data—e.g. participant privacy or use of data from a third party—those must be specified.

Reviewer #1: Yes

Reviewer #2: Yes

4. Is the manuscript presented in an intelligible fashion and written in standard English?

PLOS ONE does not copyedit accepted manuscripts, so the language in submitted articles must be clear, correct, and unambiguous. Any typographical or grammatical errors should be corrected at revision, so please note any specific errors here.

Reviewer #1: Yes

Reviewer #2: Yes

5. Review Comments to the Author

Please use the space provided to explain your answers to the questions above. You may also include additional comments for the author, including concerns about dual publication, research ethics, or publication ethics. (Please upload your review as an attachment if it exceeds 20,000 characters)

Reviewer #1: 1. Introduction (Section)

Limited biological interpretation

• Findings are described, but biological or clinical implications are not deeply explored.

• The “receptor- and sensing-associated program” is vague and not explained.

• Briefly explain:

o What these programs represent biologically

o Why spatial heterogeneity matters in DRG

-> We thank the reviewer for this helpful comment. We agree that the biological interpretation of the identified metaprograms, particularly IMM7_P4, required additional clarification. In the revised manuscript, we expanded both the Results and Discussion sections to provide a more detailed biological interpretation of the receptor- and sensing-associated program.

Specifically, we now describe IMM7_P4 as a transcriptional program enriched for genes associated with sensory signaling and receptor-mediated communication, including IGFBP7, HRH1, SCN9A, P2RY1, CALCA, RET, and related signaling components. Rather than representing a classical pro-inflammatory immune state, this program appears to reflect a receptor- and sensory-interaction-associated transcriptional axis within the DRG immune microenvironment. We have revised the terminology and discussion accordingly to improve biological clarity.

We also expanded the discussion of spatial heterogeneity in the DRG. The revised manuscript now emphasizes that immune organization within the DRG is not uniformly distributed throughout the tissue but instead exhibits localized micro-niche architecture. Because the DRG represents a specialized neuroimmune interface where neuronal and immune elements coexist in close anatomical proximity, spatial positioning may provide an additional layer of biological organization beyond transcriptional identity alone. We therefore discuss how localized enrichment of specific transcriptional programs may contribute to regionally distinct immune microenvironments within the ganglion.

The relevant additions have been incorporated into the Introduction, Results, and Discussion sections of the revised manuscript.

2. Results (Section)

Lack of quantitative highlights

• No numbers summarizing key results (e.g., % variance explained, effect sizes, significance levels). Include 1–2 key metrics (e.g., significance, clustering strength, enrichment scores).

-> We thank the reviewer for this suggestion. We agree that the original Results section emphasized qualitative descriptions and would benefit from the inclusion of key quantitative metrics.

In the revised manuscript, we incorporated additional numerical summaries throughout the Results section. Specifically, we now report quantitative measures associated with the NMF-derived metaprograms, pathway enrichment analyses, and spatial clustering results. For the spatial analyses, we added region-specific clustering statistics derived from permutation-based nearest-neighbor analyses. Across all four Xenium regions, IMM7_P4-high cells exhibited substantially reduced nearest-neighbor distances compared with randomly sampled cells of equal number, with clustering ratios ranging from 0.515 to 0.634 and permutation-based P values of 0.001 in all regions.

We additionally incorporated quantitative measures supporting projection robustness. Leave-one-gene-out analyses demonstrated strong preservation of IMM7_P4 scores (Pearson r = 0.914–0.999), while random 10% gene-dropout analyses across 1,000 iterations maintained high concordance with the original projection (mean Pearson r = 0.960; 95% interval = 0.883–0.992). Top-decile P4-high classification also remained stable (mean overlap = 0.824; mean Jaccard index = 0.713).

These additions provide quantitative support for the reproducibility, stability, and spatial organization of the identified immune metaprograms and have been incorporated throughout the revised Results section.

3. Discussion (Section)

• Make the impact explicit:

o How this informs pain mechanisms

o How it may influence targeted therapies

-> We thank the reviewer for this important suggestion. We agree that the broader implications of the findings for pain biology and DRG-targeted interventions should be more clearly articulated.

In the revised Discussion, we expanded the interpretation of the identified immune metaprograms within the context of neuroimmune regulation in the dorsal root ganglion. Specifically, we now emphasize that the observed transcriptional architecture does not support a model of uniform ganglion-wide immune activation. Instead, our findings suggest that immune activity within the human DRG may be organized into spatially localized transcriptional micro-niches composed of distinct but coexisting immune programs. This framework provides a biologically grounded perspective for understanding how neuroimmune interactions may occur in anatomically restricted regions of the ganglion rather than through a single dominant inflammatory state.

We also expanded the discussion regarding potential relevance to pain mechanisms. Although the present study does not establish causal relationships between individual metaprograms and pain phenotypes, the identification of receptor- and sensing-associated immune programs suggests that localized immune states may participate in region-specific neuroimmune communication within the DRG. We therefore discuss how transcriptionally distinct immune microenvironments could contribute to heterogeneity in sensory signaling and neuroimmune regulation.

Regarding therapeutic implications, we intentionally maintained a conservative interpretation. We do not claim that any identified metaprogram directly predicts treatment response or constitutes a therapeutic target. However, the findings provide biological context for DRG-targeted interventions by suggesting that such interventions are applied within a spatially heterogeneous immune microenvironment rather than a uniformly inflamed tissue compartment. We have therefore revised the Discussion to clarify that the present study generates a framework for future mechanistic and translational investigations rather than evidence of therapeutic efficacy.

These additions have been incorporated into the revised Discussion section.

4. Conclusion (Section)

• Final sentence is descriptive rather than impactful.

• Does not clearly state why this matters for patients or interventions.

• Explicitly state what is new compared to prior DRG studies.

-> We thank the reviewer for this valuable suggestion. We agree that the original Conclusion primarily summarized the findings and did not sufficiently emphasize their broader significance or novelty.

In the revised manuscript, we strengthened the Conclusion to more clearly highlight the principal contribution of the study. Specifically, we now emphasize that, unlike previous DRG studies that primarily focused on cataloging cell types or describing gene-expression profiles, the present work identifies reproducible immune transcriptional metaprograms and demonstrates their spatial organization within human DRG tissue. Our findings support a model in which immune activity is organized as localized micro-niches rather than as a uniformly distributed inflammatory state.

We further clarified the potential relevance of this framework for understanding neuroimmune interactions within the DRG. Although the study does not establish therapeutic efficacy or causal mechanisms of pain, it provides a spatially informed perspective on immune organization in a clinically important target of pain interventions. We therefore emphasize that the principal advance of the study is the integration of transcriptional program discovery with spatial organization, providing a quantitative framework for future investigations of DRG biology and neuroimmune regulation.

The Conclusion section has been revised accordingly to better articulate the novelty, significance, and potential translational relevance of the findings.

Reviewer #2: The study objectives are well aligned with the methodology; it focuses to move beyond CNS-centric immune models and determining if DRG immune activity reflects a single inflammatory axis or heterogeneous micro-niches. The authors clearly defined the transcriptional decomposition (NMF) to spatial projection (Xenium) to test the hypothesis of anatomical micro-niches. However the following scientific gaps and data errors need to be addressed:

The present study design is descriptive and observational. While it identifies a "receptor-enriched" program (IMM7_P4), it fails to explain how these cells functionally interact with neurons or contribute to pain signalling mechanism.

-> We thank the reviewer for this important comment and agree that the present study is descriptive and observational in nature. Our primary objective was not to establish functional neuron–immune interactions or causal mechanisms of pain signaling, but rather to determine whether immune transcriptional states within the human DRG are organized as a single dominant inflammatory axis or as spatially localized transcriptional micro-niches.

We agree that the original manuscript did not sufficiently emphasize this distinction. In the revised Discussion, we have clarified that the identified IMM7_P4 program should not be interpreted as evidence of a direct neuron–immune signaling mechanism or a causal contributor to pain. Instead, IMM7_P4 is interpreted as a receptor- and sensing-associated transcriptional state identified through unsupervised decomposition of immune nuclei.

To address the reviewer’s concern, we expanded the Discussion to explicitly state that the present findings generate hypotheses regarding potential neuroimmune interactions but do not demonstrate functional communication between immune cells and neurons. We further emphasize that future studies incorporating higher-resolution spatial profiling, ligand–receptor analyses, functional assays, and disease-associated DRG specimens will be necessary to determine whether specific immune metaprograms participate directly in pain-related signaling pathways.

Accordingly, the revised manuscript now presents the identified metaprograms as a framework for future mechanistic investigation rather than as evidence of established neuron–immune signaling mechanisms.

The study does not correlate with available clinal data. For example, how this study could extrapolate to conditions like chronic pain, age, or sex of the donors.

-> We thank the reviewer for this important observation and agree that age, sex, and disease-associated clinical variables represent biologically relevant factors that may influence immune organization within the DRG.

However, the primary objective of the present study was to characterize the baseline transcriptional and spatial architecture of immune states within human DRG tissue rather than to evaluate clinical correlates or disease-specific mechanisms. The publicly available datasets used in this study provide limited and heterogeneous clinical metadata, and the available sample size is insufficient to support statistically meaningful stratification according to age, sex, or chronic pain status.

We therefore agree that direct extrapolation of the identified metaprograms to chronic pain phenotypes, aging-related processes, or sex-specific immune organization is not currently supported by the available data. To address this concern, we have revised the Discussion and Limitations sections to explicitly acknowledge this limitation and to clarify that the present findings should be interpreted as a reference framework describing immune organization in human DRG tissue.

We additionally note that future studies incorporating larger cohorts, disease-associated DRG specimens, and richer clinical annotation will be necessary to determine whether specific immune metaprograms are associated with chronic pain, aging, sex-related biological variation, or therapeutic response. We have added this point to the revised manuscript.

The Micro-niches do not explicitly define these niches in relation to known DRG landmarks, such as proximity to satellite glial cells, specific neuronal subtypes (e.g., Aβ vs. C-fibres).

-> We thank the reviewer for this important observation. We agree that the term “micro-niche” may imply a more explicit anatomical definition than was directly demonstrated in the original manuscript.

In the present study, the term micro-niche was used operationally to describe localized spatial enrichment of transcriptionally defined immune metaprograms within DRG tissue. Our analyses demonstrate non-random spatial aggregation of metaprogram-high cells, but they do not directly establish the cellular or anatomical components that define these regions. Specifically, the current study was not designed to determine proximity relationships between immune metaprograms and particular DRG landmarks such as satellite glial cells, Schwann cells, Aβ neurons, or C-fiber neurons.

We therefore agree that the identified micro-niches should be interpreted as spatially localized transcriptional domains rather than anatomically resolved cellular niches. To address this concern, we have revised the Discussion and Limitations sections to clarify this distinction and to explicitly state that higher-resolution spatial analyses incorporating broader marker coverage will be required to determine the cellular composition and anatomical context of these regions.

Accordingly, the revised manuscript now defines micro-niches as localized areas of metaprogram enrichment rather than as fully characterized cellular interaction domains.

The exclusion of approx. 90% of the initial population due to "neuronal contamination suggests significant technical limitations in the discovery dataset (GSE189501). This may bias the remaining genuine nuclei toward specific survival. Also justify whether this exclusion introduces a selection bias toward certain immune lineages. The authors state NMF was not used to define subtypes, it would be beneficial to show which broad immune lineages (from the 27.3% that were assigned) contribute most heavily to each program.

-> We thank the reviewer for this thoughtful comment. We agree that the substantial reduction from the initial immune candidate population to the final curated TRUE immune population warrants additional clarification.

The initial immune candidate set was intentionally defined with high sensitivity in order to minimize false exclusion of potentially immune-associated nuclei. As a consequence, a substantial number of nuclei exhibiting residual neuronal signatures were retained during the initial screening stage. The subsequent filtering step was therefore designed to remove cells with evidence of neuronal contamination rather than to enrich for specific immune phenotypes. We have clarified this rationale in the revised Methods and Supplementary Information.

We agree that any filtering strategy has the potential to introduce selection bias. To address this concern, we performed additional characterization of the curated TRUE immune population and now provide a program-by-lineage composition analysis. Using broad immune lineage annotations (CD4 T cells, CD8 T cells, NK cells, B cells, plasma cells, monocytes, macrophages, dendritic cells, and unassigned cells), we evaluated the dominant program assignment of each nucleus and visualized lineage contributions across all seven metaprograms.

Importantly, these analyses demonstrate that the identified metaprograms do not correspond directly to individual immune lineages. Instead, multiple lineage categories contribute to each program, supporting our interpretation that the NMF-derived components represent transcriptional programs rather than refined immune subtypes. We have added these results as supplementary analyses and expanded the corresponding discussion in the revised manuscript.

We additionally clarify that the high proportion of unassigned cells reflects the restricted panel97 feature space, which lacks many canonical subtype-defining immune markers. Consequently, the elevated NA fraction should not be interpreted as evidence of biologically undefined immune populations, but rather as a limitation of subtype assignment under a constrained gene panel.

These clarifications have been incorporated into the revised Methods, Results, and Supplementary sections.

The spatial projection depends on a 97-gene panel. Identifying complex "metaprograms" using such a small subset of the transcriptome may lead to false positives in spatial clustering, as many genes in the panel may be shared across multiple biological processes.

-> We thank the reviewer for this important comment and agree that projection of transcriptional programs within a restricted gene panel requires careful evaluation. The limited panel97 feature space is an inherent constraint of the Xenium dataset and represents an important consideration when interpreting spatial projection results.

To directly address this concern, we performed additional robustness analyses for the IMM7_P4 metaprogram. First, we conducted leave-one-gene-out analyses across all 30 genes comprising the IMM7_P4 signature. Recalculated scores remained highly concordant with the original projection, with Pearson correlation coefficients ranging from 0.914 to 0.999. Second, we performed random gene-dropout analyses in which 10% of signature genes were randomly removed across 1,000 independent iterations. Despite repeated perturbation of the signature, projection scores remained highly stable (mean Pearson r = 0.960; 95% interval = 0.883–0.992; mean Spearman r = 0.956).

To further evaluate classification stability, we assessed preservation of the original P4-high population following random gene dropout. Top-decile P4-high assignments remained largely conserved (mean overlap = 0.824; mean Jaccard index = 0.713), indicating that the projected spatial pattern was not driven by a small number of individual genes.

These additional analyses suggest that the observed spatial clustering is not dependent on any single marker gene and is robust to perturbation of the underlying signature. We have incorporated these analyses into the revised Methods, Results, and Supplementary Information and explicitly acknowledge the limitations associated with the restricted panel97 feature space.

Justify the selection k=7 programs for a dataset of only 388 cells. NMF requires high cell-to-rank ratios to avoid capturing noise as reproducible programs.

-> We thank the reviewer for raising this important methodological concern. We agree that NMF rank selection requires caution, particularly when applied to a relatively small curated immune population.

In the revised manuscript, we expanded the Methods and Results sections to clarify that k = 7 was not selected arbitrarily. Candidate ranks from k = 4 to k = 9 were evaluated using nmfEstimateRank with 30 independent runs per rank. Rank selection was based on multiple stability criteria, including cophenetic correlation, dispersion, silhouette index, and consensus matrix structure. The k = 7 solution showed stable separation of transcriptional programs without the fragmentation observed at higher ranks.

We also clarified that the analysis was not intended to define discrete immune subtypes. Instead, NMF was used as a dimensional decomposition method to identify co-expressed transcriptional axes within a restricted, spatially projectable gene panel. With 388 TRUE immune nuclei and k = 7, the effective cell-to-rank ratio was approximately 55 cells per component, which we considered sufficient for exploratory program-level decomposition when supported by rank stability metrics and biological interpretability.

To reduce the possibility that the identified programs reflected noise, we further characterized the k = 7 solution using gene-loading patterns, program-level differential expression, pathway enrichment, broad lineage composition, external dataset projection, and spatial aggregation analyses. These analyses showed that the programs were not isolated numerical artifacts but reproducible transcriptional structures with interpretable biological and spatial properties.

We have revised the manuscript to make this justification explicit and to acknowledge that the metaprograms should be interpreted as stable transcriptional representations within the current analytical framework rather than definitive immune cell categories.

The label for IMM7_P4 is quite broad. Provide a more specific analysis of the top-loading genes (e.g., IGFBP7, HRH1) to clarify what specific signaling pathways (e.g., histaminergic, growth factor) in the revised manuscript.

-> We thank the reviewer for this helpful suggestion. We agree that the original label “receptor- and sensing-associated program” was intentionally conservative but did not sufficiently convey the biological features represented by the highest-loading genes within IMM7_P4.

To address this concern, we performed additional examination of the top-loading genes and expanded the biological interpretation of the program in the revised manuscript. The highest-loading genes included IGFBP7, HRH1, HRH2, P2RY1, SCN9A, CALCA, RET, GFRA2, IL31RA, EDNRA, and CHRNA7. These genes collectively suggest enrichment of multiple signaling systems associated with receptor-mediated environmental sensing and neuroimmune communication.

Specifically, HRH1 and HRH2 are components of histaminergic signaling pathways, P2RY1 is involved in purinergic signaling, RET and GFRA2 participate in neurotrophic signaling, IGFBP7 is associated with growth factor–related signaling, and SCN9A and CALCA are linked to sensory and neuropeptide signaling processes. Rather than representing a single pathway, IMM7_P4 appears to capture a convergent signaling state characterized by receptor-mediated environmental responsiveness and sensory-associated signaling features.

We therefore revised the Results and Discussion sections to provide a more detailed biological interpretation of IMM7_P4 and to explicitly discuss the signaling systems represented by the highest-loading genes. At the same time, we retained a cautious interpretation because the present study does not directly measure pathway activity or functional signaling interactions.

For the nearest-neighbour distance analysis, 200 permutations is a relatively low number for establishing high-confidence spatial significance. Increasing the permutation numbers to 5 times or more would strengthen the statistical significance.

-> We thank the reviewer for this helpful suggestion. We agree that increasing the number of permutations strengthens the statistical assessment of spatial aggregation.

In the revised analysis, we repeated the nearest-neighbor distance permutation testing using 1,000 permutations, corresponding to a five-fold increase from the original 200 permutations. The spatial clustering results remained significant across all four Xenium regions. IMM7_P4-high cells showed reduced nearest-neighbor distances compared with randomly sampled cells of equal number, with clustering ratios ranging from 0.515 to 0.634 and permutation-based P values of 0.001 in all four regions.

These updated analyses have been incorporated into the revised Methods and Results sections, and the corresponding spatial clustering summary has been updated in the revised manuscript.

Supplementary Figure 1 shows that 72.7% of cells remained unassigned to a subtype (NA). Discuss how this lack of lineage resolution impacts the interpretation of metaprograms as "functional states" rather than merely unidentified cell types.

-> We thank the reviewer for this important comment. We agree that the high proportion of unassigned cells requires careful interpretation and clarification.

As shown in Supplementary Figure 1, the elevated NA fraction primarily reflects the restricted panel97 feature space used for projection and subtype annotation. Many canonical immune lineage markers are absent from the Xenium-derived panel97 gene set, substantially limiting the ability to assign fine immune subtypes using conventional marker-based approaches. Consequently, the large proportion of unassigned cells should not be interpreted as evidence for biologically undefined cell populations, but rather as a limitation of subtype classification within a constrained feature space.

To address this concern, we performed additional analyses examining the contribution of broad immune lineage categories to each metaprogram. These analyses demonstrated that multiple lineage categories contribute to individual metaprograms and that no metaprogram corresponds uniquely to a single immune subtype. This observation supports the interpretation that the identified NMF components represent transcriptional programs that cut across lineage boundaries rather than refined cell-type clusters.

We have revised the Methods, Results, and Discussion sections to clarify this distinction. Specifically, we now emphasize that the metaprograms should be interpreted as functional transcriptional states identified through unsupervised decomposition of the available feature space, while acknowledging that the restricted panel97 gene set limits definitive lineage assignment. We further discuss this issue in the Limitations section and note that future studies using broader transcriptomic coverage will be required to resolve the relationship between these programs and canonical immune cell identities more precisely.

In the validation using GSE168243, provide a quantitative correlation matrix or Jaccard index between the gene signatures in the discovery and validation cohorts to move beyond the visual comparable architecture claim.

-> We thank the reviewer for this helpful suggestion. We agree that the original validation analysis relied primarily on visual comparison of projected program architecture and required additional quantitative support.

To address this point, we recalculated the validation analysis in GSE168243 using the IMM7 top-gene signatures derived from the discovery cohort. Seven IMM7 programs were projected onto 1,496 nuclei from six validation samples. On average, 26.8 of 30 signature genes per program were detected and used for scoring in the validation dataset.

We then quantified the projected metaprogram architecture using program-level correlation and top-decile overlap metrics. The validation dataset showed strong program-level correlation structure, with a mean absolute cell-level Spearman correlation of 0.855 and a mean absolute sample-level Spearman correlation of 0.902 across IMM7 programs. We also calculated pairwise top-decile Jaccard similarity among program-high populations, which yielded a mean off-diagonal Jaccard index of 0.575. In addition, the dominant-program composition remained heterogeneous across validation samples, with a mean composition entropy of 1.42.

These quantitative metrics have been added to the revised Supplementary Information and strengthen the conclusion that the inferred IMM7 metaprogram architecture is detectable in an independent human DRG snRNA-seq dataset, beyond visual similarity alone.

The inter-individual variability parameter indicates that the spatial results are based on limited tissue regions. Justify whether the micro-niche patterns observed are consistent across different human donors.

-> We thank the reviewer for this important comment and agree that inter-individual variability represents an important consideration when interpreting spatial transcriptomic findings.

The publicly available Xenium dataset used in this study contains a limited number of human DRG tissue regions, which restricts our ability to perform a comprehensive donor-level analysis. We therefore agree that the current dataset is not sufficient to establish the prevalence of specific micro-niche patterns across the broader human population.

To address this concern, we expanded the spatial aggregation analysis across all four available Xenium regions and quantified IMM7_P4 clustering independently in each region using 1,000-permutation nearest-neighbor analyses. Importantly, significant spatial aggregation was consistently observed in all four regions. Clustering ratios ranged from 0.515 to 0.634, and permutation-based P values were 0.001 in every region. These findings indicate that the observed micro-niche organization is reproducible across independent tissue regions rather than being driven by a single localized observation.

We have revised the Discussion and Limitations sections to clarify that our findings demonstrate regional reproducibility within the available dataset but do not establish population-level generalizability. Larger studies incorporating additional donors and clinically annotated specimens will be required to determine the extent of inter-individual variation in DRG immune micro-niche organization.

Accordingly, we now interpret the observed spatial organization as a reproducible feature across the available DRG regions while acknowledging that broader donor-level validation remains an important direction for future investigation.

In Figure 4, include overlay computational data to show where P4-high cells sit in relation to DRG neurons.

-> We thank the reviewer for this constructive suggestion. We agree that additional anatomical context would be helpful when interpreting the spatial organization of the IMM7_P4 program.

The primary objective of Figure 4 was to determine whether IMM7_P4-high cells exhibit non-random spatial aggregation within the human DRG. Accordingly, our analyses focused on spatial clustering and micro-niche organization rather than on defining direct relationships with specific neuronal populations. We note that the Xenium panel used in this study contains a restricted set of genes and was not specifically designed to comprehensively classify neuronal subtypes or resolve detailed neuron–immune spatial interactions.

To address the reviewer’s concern, we expanded the Discussion to clarify that the identified micro-niches represent localized regions of metaprogram enrichment rather than anatomically defined neuron-associated niches. We also explicitly acknowledge that the current dataset does not permit robust assignment of IMM7_P4-enriched regions to specific neuronal subtypes, satellite glial cells, or other anatomical landmarks within the DRG.

We agree that systematic characterization of the spatial relationship between immune metaprograms and defined neuronal populations represents an important future direction. However, we believe that such analyses would require broader marker coverage and higher-resolution neuroanatomical annotation than is available within the current Xenium panel. We have therefore revised the manuscript to clarify the scope of our conclusions and to discuss this limitation explicitly.

Revise the citations section and ensure that they are chronologically consistent. The manuscript mentions data retrieval in 2025/2026 but includes references that are seemingly future-dated relative to current scientific literature (e.g., bioRxiv 2025, Nature 2025)

-> We thank the reviewer for identifying this issue. We carefully re-examined the reference list, with particular attention to preprints and citations dated 2025 or 2026, to ensure that the bibliography is chronologically consistent and reflects the most accurate publication status currently available.

Specifically, we reviewed the references that were most likely to raise concerns regarding future-dated or outdated citation metadata. The citation to Bhuiyan et al., “A reference atlas of the human dorsal root ganglion,” remains available as a bioRxiv preprint, and we did not identify a corresponding peer-reviewed journal version at the time of revision. We therefore retained this citation as a preprint while ensuring that the citation information is clearly labeled and accurate.

We also verified the citation to Wang et al., “An enteric-DRG pathway for interoception and visceral pain in mice.” This article has been formally published in Neuron as a 2026 article, with the final citation listed as Neuron 114(1):105–121.e6. Therefore, this reference was retained as a published article rather than treated as an erroneous future-dated citation.

In contrast, we found that the previous citation to Boe et al., “Spatial transcriptomics reveals influence of microenvironment on intrinsic fates in melanoma therapy resistance,” which had been cited as a 2024 bioRxiv preprint, now has a peer-reviewed published version in Genome Biology. We updated this reference accordingly and replaced the preprint citation with the published journal citation.

In addition to these specific corrections, we reviewed the full reference list for consistency between publication year, citation status, and manuscript timeline. References with incorrect or outdated metadata were corrected, and published versions were cited whenever available. The revised reference list has been updated accordingly.
