# Supplementary material for: Spatially localized immune metaprograms reveal micro-niche organization in the human Dorsal Root Ganglion
Source: PLoS One. 2026 Aug 3;21(8):e0354750. doi: 10.1371/journal.pone.0354750 (PMC13432135; doi:10.1371/journal.pone.0354750)
Supplement: Peer review history

# Peer review: Decision Letter 0

**DOI:** 10.1371/journal.pone.0354750.r001

**Authors:** Kah Hui Wong

4 May 2026

PONE-D-26-12553

Spatially localized immune metaprograms reveal micro-niche organization in the human dorsal root ganglion

PLOS One

Dear Dr. Kim,

Thank you for submitting your manuscript to PLOS ONE. After careful consideration, we feel that it has merit but does not fully meet PLOS ONE’s publication criteria as it currently stands. Therefore, we invite you to submit a revised version of the manuscript that addresses the points raised during the review process.

Please submit your revised manuscript by Jun 18 2026 11:59PM. If you will need more time than this to complete your revisions, please reply to this message or contact the journal office at plosone@plos.org. When you're ready to submit your revision, log on to https://www.editorialmanager.com/pone/ and select the 'Submissions Needing Revision' folder to locate your manuscript file.

Please include the following items when submitting your revised manuscript:

- A letter that responds to each point raised by the academic editor and reviewer(s). You should upload this letter as a separate file labeled 'Response to Reviewers'.
- A marked-up copy of your manuscript that highlights changes made to the original version. You should upload this as a separate file labeled 'Revised Manuscript with Track Changes'.
- An unmarked version of your revised paper without tracked changes. You should upload this as a separate file labeled 'Manuscript'.

If you would like to make changes to your financial disclosure, please include your updated statement in your cover letter. Guidelines for resubmitting your figure files are available below the reviewer comments at the end of this letter.

If applicable, we recommend that you deposit your laboratory protocols in protocols.io to enhance the reproducibility of your results. Protocols.io assigns your protocol its own identifier (DOI) so that it can be cited independently in the future. For instructions see: https://journals.plos.org/plosone/s/submission-guidelines#loc-laboratory-protocols. Additionally, PLOS ONE offers an option for publishing peer-reviewed Lab Protocol articles, which describe protocols hosted on protocols.io. Read more information on sharing protocols at https://plos.org/protocols?utm_medium=editorial-email&utm_source=authorletters&utm_campaign=protocols.

As the corresponding author, your ORCID iD is verified in the submission system and will appear in the published article. PLOS supports the use of ORCID, and we encourage all coauthors to register for an ORCID iD and use it as well. Please encourage your coauthors to verify their ORCID iD within the submission system before final acceptance, as unverified ORCID iDs will not appear in the published article. *Only*  the individual author can complete the verification step; PLOS staff *cannot*  verify ORCID iDs on behalf of authors.

We look forward to receiving your revised manuscript.

Kind regards,

Kah Hui Wong, Ph.D.

Academic Editor

PLOS One

Journal Requirements:

When submitting your revision, we need you to address these additional requirements.

1. Please ensure that your manuscript meets PLOS ONE's style requirements, including those for file naming. The PLOS ONE style templates can be found at

https://journals.plos.org/plosone/s/file?id=wjVg/PLOSOne_formatting_sample_main_body.pdf and

https://journals.plos.org/plosone/s/file?id=ba62/PLOSOne_formatting_sample_title_authors_affiliations.pdf.

2. Thank you for stating in your Funding Statement:

“This work was supported by the National Research Foundation of Korea (NRF) funded by the Ministry of Science and ICT (2019R1A5A2027588 and 2019M3E5D3073104 to T.K.). “

Please provide an amended statement that declares *all* the funding or sources of support (whether external or internal to your organization) received during this study, as detailed online in our guide for authors at http://journals.plos.org/plosone/s/submit-now.  Please also include the statement “There was no additional external funding received for this study.” in your updated Funding Statement.

Please include your amended Funding Statement within your cover letter. We will change the online submission form on your behalf.

3. Thank you for stating the following financial disclosure:

“This work was supported by the National Research Foundation of Korea (NRF) funded by the Ministry of Science and ICT (2019R1A5A2027588 and 2019M3E5D3073104 to T.K.). “

Please state what role the funders took in the study.  If the funders had no role, please state: "The funders had no role in study design, data collection and analysis, decision to publish, or preparation of the manuscript."

If this statement is not correct you must amend it as needed.

Please include this amended Role of Funder statement in your cover letter; we will change the online submission form on your behalf.

4. PLOS requires an ORCID iD for the corresponding author in Editorial Manager on papers submitted after December 6th, 2016. Please ensure that you have an ORCID iD and that it is validated in Editorial Manager. To do this, go to ‘Update my Information’ (in the upper left-hand corner of the main menu), and click on the Fetch/Validate link next to the ORCID field. This will take you to the ORCID site and allow you to create a new iD or authenticate a pre-existing iD in Editorial Manager.

5. Please be informed that funding information should not appear in the Acknowledgments section or other areas of your manuscript. We will only publish funding information present in the Funding Statement section of the online submission form. Please remove any funding-related text from the manuscript.

6.We notice that your supplementary figures are included in the manuscript file. Please remove them and upload them with the file type 'Supporting Information'. Please ensure that each Supporting Information file has a legend listed in the manuscript after the references list

7. If the reviewer comments include a recommendation to cite specific previously published works, please review and evaluate these publications to determine whether they are relevant and should be cited. There is no requirement to cite these works unless the editor has indicated otherwise.

Additional Editor Comments (if provided):

Dear Dr Tae-Min Kim,

The manuscript titled “Spatially localized immune metaprograms reveal micro-niche organization in the human dorsal root ganglion” has been evaluated by two reviewers.

Please address the comments and concerns raised by the reviewers and highlight the changes in the revised manuscript.

Thank you

Kah Hui Wong

[Note: HTML markup is below. Please do not edit.]

Reviewers' comments:

Reviewer's Responses to Questions

**Comments to the Author**

1. Is the manuscript technically sound, and do the data support the conclusions?

The manuscript must describe a technically sound piece of scientific research with data that supports the conclusions. Experiments must have been conducted rigorously, with appropriate controls, replication, and sample sizes. The conclusions must be drawn appropriately based on the data presented.

Reviewer #1: Yes

Reviewer #2: Yes

**********

2. Has the statistical analysis been performed appropriately and rigorously?

Reviewer #1: No

Reviewer #2: Yes

**********

3. Have the authors made all data underlying the findings in their manuscript fully available?

The PLOS Data policy requires authors to make all data underlying the findings described in their manuscript fully available without restriction, with rare exception (please refer to the Data Availability Statement in the manuscript PDF file). The data should be provided as part of the manuscript or its supporting information, or deposited to a public repository. For example, in addition to summary statistics, the data points behind means, medians and variance measures should be available. If there are restrictions on publicly sharing data—e.g. participant privacy or use of data from a third party—those must be specified.

Reviewer #1: Yes

Reviewer #2: Yes

**********

4. Is the manuscript presented in an intelligible fashion and written in standard English?

PLOS ONE does not copyedit accepted manuscripts, so the language in submitted articles must be clear, correct, and unambiguous. Any typographical or grammatical errors should be corrected at revision, so please note any specific errors here.

Reviewer #1: Yes

Reviewer #2: Yes

**********

5. Review Comments to the Author

Please use the space provided to explain your answers to the questions above. You may also include additional comments for the author, including concerns about dual publication, research ethics, or publication ethics. (Please upload your review as an attachment if it exceeds 20,000 characters)

Reviewer #1: 1. Introduction (Section)

Limited biological interpretation

• Findings are described, but biological or clinical implications are not deeply explored.

• The “receptor- and sensing-associated program” is vague and not explained.

• Briefly explain:

o What these programs represent biologically

o Why spatial heterogeneity matters in DRG

2. Results (Section)

Lack of quantitative highlights

• No numbers summarizing key results (e.g., % variance explained, effect sizes, significance levels). Include 1–2 key metrics (e.g., significance, clustering strength, enrichment scores).

3. Discussion (Section)

• Make the impact explicit:

o How this informs pain mechanisms

o How it may influence targeted therapies

4. Conclusion (Section)

• Final sentence is descriptive rather than impactful.

• Does not clearly state why this matters for patients or interventions.

• Explicitly state what is new compared to prior DRG studies.

Reviewer #2: The study objectives are well aligned with the methodology; it focuses to move beyond CNS-centric immune models and determining if DRG immune activity reflects a single inflammatory axis or heterogeneous micro-niches. The authors clearly defined the transcriptional decomposition (NMF) to spatial projection (Xenium) to test the hypothesis of anatomical micro-niches. However the following scientific gaps and data errors need to be addressed:

The present study design is descriptive and observational. While it identifies a "receptor-enriched" program (IMM7_P4), it fails to explain how these cells functionally interact with neurons or contribute to pain signalling mechanism.

The study does not correlate with available clinal data. For example, how this study could extrapolate to conditions like chronic pain, age, or sex of the donors.

The Micro-niches do not explicitly define these niches in relation to known DRG landmarks, such as proximity to satellite glial cells, specific neuronal subtypes (e.g., Aβ vs. C-fibres).

The exclusion of approx. 90% of the initial population due to "neuronal contamination suggests significant technical limitations in the discovery dataset (GSE189501). This may bias the remaining genuine nuclei toward specific survival. Also justify whether this exclusion introduces a selection bias toward certain immune lineages. The authors state NMF was not used to define subtypes, it would be beneficial to show which broad immune lineages (from the 27.3% that were assigned) contribute most heavily to each program.

The spatial projection depends on a 97-gene panel. Identifying complex "metaprograms" using such a small subset of the transcriptome may lead to false positives in spatial clustering, as many genes in the panel may be shared across multiple biological processes.

Justify the selection k=7 programs for a dataset of only 388 cells. NMF requires high cell-to-rank ratios to avoid capturing noise as reproducible programs.

The label for IMM7_P4 is quite broad. Provide a more specific analysis of the top-loading genes (e.g., IGFBP7, HRH1) to clarify what specific signaling pathways (e.g., histaminergic, growth factor) in the revised manuscript.

For the nearest-neighbour distance analysis, 200 permutations is a relatively low number for establishing high-confidence spatial significance. Increasing the permutation numbers to 5 times or more would strengthen the statistical significance.

Supplementary Figure 1 shows that 72.7% of cells remained unassigned to a subtype (NA). Discuss how this lack of lineage resolution impacts the interpretation of metaprograms as "functional states" rather than merely unidentified cell types.

In the validation using GSE168243, provide a quantitative correlation matrix or Jaccard index between the gene signatures in the discovery and validation cohorts to move beyond the visual comparable architecture claim.

The inter-individual variability parameter indicates that the spatial results are based on limited tissue regions. Justify whether the micro-niche patterns observed are consistent across different human donors.

In Figure 4, include overlay computational data to show where P4-high cells sit in relation to DRG neurons.

Revise the citations section and ensure that they are chronologically consistent. The manuscript mentions data retrieval in 2025/2026 but includes references that are seemingly future-dated relative to current scientific literature (e.g., bioRxiv 2025, Nature 2025)

**********

6. PLOS authors have the option to publish the peer review history of their article (what does this mean?). If published, this will include your full peer review and any attached files.

If you choose “no”, your identity will remain anonymous but your review may still be made public.

**Do you want your identity to be public for this peer review?**  For information about this choice, including consent withdrawal, please see our Privacy Policy.

Reviewer #1: **Yes:** saara

Reviewer #2: No

**********

[NOTE: If reviewer comments were submitted as an attachment file, they will be attached to this email and accessible via the submission site. Please log into your account, locate the manuscript record, and check for the action link "View Attachments". If this link does not appear, there are no attachment files.]

To ensure your figures meet our technical requirements, please review our figure guidelines: https://journals.plos.org/plosone/s/figures

You may also use PLOS’s free figure tool, NAAS, to help you prepare publication quality figures: https://journals.plos.org/plosone/s/figures#loc-tools-for-figure-preparation.

NAAS will assess whether your figures meet our technical requirements by comparing each figure against our figure specifications.

Attachment Submitted filename: Reviewer`s comments.docx

# Author comment: Author response to Decision Letter 1

**DOI:** 10.1371/journal.pone.0354750.r002

17 Jun 2026

Reviewers' comments:

Reviewer's Responses to Questions

Comments to the Author

1. Is the manuscript technically sound, and do the data support the conclusions?

The manuscript must describe a technically sound piece of scientific research with data that supports the conclusions. Experiments must have been conducted rigorously, with appropriate controls, replication, and sample sizes. The conclusions must be drawn appropriately based on the data presented.

Reviewer #1: Yes

Reviewer #2: Yes

2. Has the statistical analysis been performed appropriately and rigorously?

Reviewer #1: No

Reviewer #2: Yes

3. Have the authors made all data underlying the findings in their manuscript fully available?

The PLOS Data policy requires authors to make all data underlying the findings described in their manuscript fully available without restriction, with rare exception (please refer to the Data Availability Statement in the manuscript PDF file). The data should be provided as part of the manuscript or its supporting information, or deposited to a public repository. For example, in addition to summary statistics, the data points behind means, medians and variance measures should be available. If there are restrictions on publicly sharing data—e.g. participant privacy or use of data from a third party—those must be specified.

Reviewer #1: Yes

Reviewer #2: Yes

4. Is the manuscript presented in an intelligible fashion and written in standard English?

PLOS ONE does not copyedit accepted manuscripts, so the language in submitted articles must be clear, correct, and unambiguous. Any typographical or grammatical errors should be corrected at revision, so please note any specific errors here.

Reviewer #1: Yes

Reviewer #2: Yes

5. Review Comments to the Author

Please use the space provided to explain your answers to the questions above. You may also include additional comments for the author, including concerns about dual publication, research ethics, or publication ethics. (Please upload your review as an attachment if it exceeds 20,000 characters)

Reviewer #1: 1. Introduction (Section)

Limited biological interpretation

• Findings are described, but biological or clinical implications are not deeply explored.

• The “receptor- and sensing-associated program” is vague and not explained.

• Briefly explain:

o What these programs represent biologically

o Why spatial heterogeneity matters in DRG

-> We thank the reviewer for this helpful comment. We agree that the biological interpretation of the identified metaprograms, particularly IMM7_P4, required additional clarification. In the revised manuscript, we expanded both the Results and Discussion sections to provide a more detailed biological interpretation of the receptor- and sensing-associated program.

Specifically, we now describe IMM7_P4 as a transcriptional program enriched for genes associated with sensory signaling and receptor-mediated communication, including IGFBP7, HRH1, SCN9A, P2RY1, CALCA, RET, and related signaling components. Rather than representing a classical pro-inflammatory immune state, this program appears to reflect a receptor- and sensory-interaction-associated transcriptional axis within the DRG immune microenvironment. We have revised the terminology and discussion accordingly to improve biological clarity.

We also expanded the discussion of spatial heterogeneity in the DRG. The revised manuscript now emphasizes that immune organization within the DRG is not uniformly distributed throughout the tissue but instead exhibits localized micro-niche architecture. Because the DRG represents a specialized neuroimmune interface where neuronal and immune elements coexist in close anatomical proximity, spatial positioning may provide an additional layer of biological organization beyond transcriptional identity alone. We therefore discuss how localized enrichment of specific transcriptional programs may contribute to regionally distinct immune microenvironments within the ganglion.

The relevant additions have been incorporated into the Introduction, Results, and Discussion sections of the revised manuscript.

2. Results (Section)

Lack of quantitative highlights

• No numbers summarizing key results (e.g., % variance explained, effect sizes, significance levels). Include 1–2 key metrics (e.g., significance, clustering strength, enrichment scores).

-> We thank the reviewer for this suggestion. We agree that the original Results section emphasized qualitative descriptions and would benefit from the inclusion of key quantitative metrics.

In the revised manuscript, we incorporated additional numerical summaries throughout the Results section. Specifically, we now report quantitative measures associated with the NMF-derived metaprograms, pathway enrichment analyses, and spatial clustering results. For the spatial analyses, we added region-specific clustering statistics derived from permutation-based nearest-neighbor analyses. Across all four Xenium regions, IMM7_P4-high cells exhibited substantially reduced nearest-neighbor distances compared with randomly sampled cells of equal number, with clustering ratios ranging from 0.515 to 0.634 and permutation-based P values of 0.001 in all regions.

We additionally incorporated quantitative measures supporting projection robustness. Leave-one-gene-out analyses demonstrated strong preservation of IMM7_P4 scores (Pearson r = 0.914–0.999), while random 10% gene-dropout analyses across 1,000 iterations maintained high concordance with the original projection (mean Pearson r = 0.960; 95% interval = 0.883–0.992). Top-decile P4-high classification also remained stable (mean overlap = 0.824; mean Jaccard index = 0.713).

These additions provide quantitative support for the reproducibility, stability, and spatial organization of the identified immune metaprograms and have been incorporated throughout the revised Results section.

3. Discussion (Section)

• Make the impact explicit:

o How this informs pain mechanisms

o How it may influence targeted therapies

-> We thank the reviewer for this important suggestion. We agree that the broader implications of the findings for pain biology and DRG-targeted interventions should be more clearly articulated.

In the revised Discussion, we expanded the interpretation of the identified immune metaprograms within the context of neuroimmune regulation in the dorsal root ganglion. Specifically, we now emphasize that the observed transcriptional architecture does not support a model of uniform ganglion-wide immune activation. Instead, our findings suggest that immune activity within the human DRG may be organized into spatially localized transcriptional micro-niches composed of distinct but coexisting immune programs. This framework provides a biologically grounded perspective for understanding how neuroimmune interactions may occur in anatomically restricted regions of the ganglion rather than through a single dominant inflammatory state.

We also expanded the discussion regarding potential relevance to pain mechanisms. Although the present study does not establish causal relationships between individual metaprograms and pain phenotypes, the identification of receptor- and sensing-associated immune programs suggests that localized immune states may participate in region-specific neuroimmune communication within the DRG. We therefore discuss how transcriptionally distinct immune microenvironments could contribute to heterogeneity in sensory signaling and neuroimmune regulation.

Regarding therapeutic implications, we intentionally maintained a conservative interpretation. We do not claim that any identified metaprogram directly predicts treatment response or constitutes a therapeutic target. However, the findings provide biological context for DRG-targeted interventions by suggesting that such interventions are applied within a spatially heterogeneous immune microenvironment rather than a uniformly inflamed tissue compartment. We have therefore revised the Discussion to clarify that the present study generates a framework for future mechanistic and translational investigations rather than evidence of therapeutic efficacy.

These additions have been incorporated into the revised Discussion section.

4. Conclusion (Section)

• Final sentence is descriptive rather than impactful.

• Does not clearly state why this matters for patients or interventions.

• Explicitly state what is new compared to prior DRG studies.

-> We thank the reviewer for this valuable suggestion. We agree that the original Conclusion primarily summarized the findings and did not sufficiently emphasize their broader significance or novelty.

In the revised manuscript, we strengthened the Conclusion to more clearly highlight the principal contribution of the study. Specifically, we now emphasize that, unlike previous DRG studies that primarily focused on cataloging cell types or describing gene-expression profiles, the present work identifies reproducible immune transcriptional metaprograms and demonstrates their spatial organization within human DRG tissue. Our findings support a model in which immune activity is organized as localized micro-niches rather than as a uniformly distributed inflammatory state.

We further clarified the potential relevance of this framework for understanding neuroimmune interactions within the DRG. Although the study does not establish therapeutic efficacy or causal mechanisms of pain, it provides a spatially informed perspective on immune organization in a clinically important target of pain interventions. We therefore emphasize that the principal advance of the study is the integration of transcriptional program discovery with spatial organization, providing a quantitative framework for future investigations of DRG biology and neuroimmune regulation.

The Conclusion section has been revised accordingly to better articulate the novelty, significance, and potential translational relevance of the findings.

Reviewer #2: The study objectives are well aligned with the methodology; it focuses to move beyond CNS-centric immune models and determining if DRG immune activity reflects a single inflammatory axis or heterogeneous micro-niches. The authors clearly defined the transcriptional decomposition (NMF) to spatial projection (Xenium) to test the hypothesis of anatomical micro-niches. However the following scientific gaps and data errors need to be addressed:

The present study design is descriptive and observational. While it identifies a "receptor-enriched" program (IMM7_P4), it fails to explain how these cells functionally interact with neurons or contribute to pain signalling mechanism.

-> We thank the reviewer for this important comment and agree that the present study is descriptive and observational in nature. Our primary objective was not to establish functional neuron–immune interactions or causal mechanisms of pain signaling, but rather to determine whether immune transcriptional states within the human DRG are organized as a single dominant inflammatory axis or as spatially localized transcriptional micro-niches.

We agree that the original manuscript did not sufficiently emphasize this distinction. In the revised Discussion, we have clarified that the identified IMM7_P4 program should not be interpreted as evidence of a direct neuron–immune signaling mechanism or a causal contributor to pain. Instead, IMM7_P4 is interpreted as a receptor- and sensing-associated transcriptional state identified through unsupervised decomposition of immune nuclei.

To address the reviewer’s concern, we expanded the Discussion to explicitly state that the present findings generate hypotheses regarding potential neuroimmune interactions but do not demonstrate functional communication between immune cells and neurons. We further emphasize that future studies incorporating higher-resolution spatial profiling, ligand–receptor analyses, functional assays, and disease-associated DRG specimens will be necessary to determine whether specific immune metaprograms participate directly in pain-related signaling pathways.

Accordingly, the revised manuscript now presents the identified metaprograms as a framework for future mechanistic investigation rather than as evidence of established neuron–immune signaling mechanisms.

The study does not correlate with available clinal data. For example, how this study could extrapolate to conditions like chronic pain, age, or sex of the donors.

-> We thank the reviewer for this important observation and agree that age, sex, and disease-associated clinical variables represent biologically relevant factors that may influence immune organization within the DRG.

However, the primary objective of the present study was to characterize the baseline transcriptional and spatial architecture of immune states within human DRG tissue rather than to evaluate clinical correlates or disease-specific mechanisms. The publicly available datasets used in this study provide limited and heterogeneous clinical metadata, and the available sample size is insufficient to support statistically meaningful stratification according to age, sex, or chronic pain status.

We therefore agree that direct extrapolation of the identified metaprograms to chronic pain phenotypes, aging-related processes, or sex-specific immune organization is not currently supported by the available data. To address this concern, we have revised the Discussion and Limitations sections to explicitly acknowledge this limitation and to clarify that the present findings should be interpreted as a reference framework describing immune organization in human DRG tissue.

We additionally note that future studies incorporating larger cohorts, disease-associated DRG specimens, and richer clinical annotation will be necessary to determine whether specific immune metaprograms are associated with chronic pain, aging, sex-related biological variation, or therapeutic response. We have added this point to the revised manuscript.

The Micro-niches do not explicitly define these niches in relation to known DRG landmarks, such as proximity to satellite glial cells, specific neuronal subtypes (e.g., Aβ vs. C-fibres).

-> We thank the reviewer for this important observation. We agree that the term “micro-niche” may imply a more explicit anatomical definition than was directly demonstrated in the original manuscript.

In the present study, the term micro-niche was used operationally to describe localized spatial enrichment of transcriptionally defined immune metaprograms within DRG tissue. Our analyses demonstrate non-random spatial aggregation of metaprogram-high cells, but they do not directly establish the cellular or anatomical components that define these regions. Specifically, the current study was not designed to determine proximity relationships between immune metaprograms and particular DRG landmarks such as satellite glial cells, Schwann cells, Aβ neurons, or C-fiber neurons.

We therefore agree that the identified micro-niches should be interpreted as spatially localized transcriptional domains rather than anatomically resolved cellular niches. To address this concern, we have revised the Discussion and Limitations sections to clarify this distinction and to explicitly state that higher-resolution spatial analyses incorporating broader marker coverage will be required to determine the cellular composition and anatomical context of these regions.

Accordingly, the revised manuscript now defines micro-niches as localized areas of metaprogram enrichment rather than as fully characterized cellular interaction domains.

The exclusion of approx. 90% of the initial population due to "neuronal contamination suggests significant technical limitations in the discovery dataset (GSE189501). This may bias the remaining genuine nuclei toward specific survival. Also justify whether this exclusion introduces a selection bias toward certain immune lineages. The authors state NMF was not used to define subtypes, it would be beneficial to show which broad immune lineages (from the 27.3% that were assigned) contribute most heavily to each program.

-> We thank the reviewer for this thoughtful comment. We agree that the substantial reduction from the initial immune ca

Attachment Submitted filename: Response to Reviewers.docx

# Peer review: Decision Letter 1

**DOI:** 10.1371/journal.pone.0354750.r003

**Authors:** Miquel Vall-llosera Camps

12 Jul 2026

Spatially localized immune metaprograms reveal micro-niche organization in the human dorsal root ganglion

PONE-D-26-12553R1

Dear Dr. Kim,

We’re pleased to inform you that your manuscript has been judged scientifically suitable for publication and will be formally accepted for publication once it meets all outstanding technical requirements.

Within one week, you’ll receive an e-mail detailing the required amendments. When these have been addressed, you’ll receive a formal acceptance letter and your manuscript will be scheduled for publication.

An invoice will be generated when your article is formally accepted. Please note, if your institution has a publishing partnership with PLOS and your article meets the relevant criteria, all or part of your publication costs will be covered. Please make sure your user information is up-to-date by logging into Editorial Manager at Editorial Manager® and clicking the ‘Update My Information' link at the top of the page. For questions related to billing, please contact billing support.

If your institution or institutions have a press office, please notify them about your upcoming paper to help maximize its impact. If they’ll be preparing press materials, please inform our press team as soon as possible -- no later than 48 hours after receiving the formal acceptance. Your manuscript will remain under strict press embargo until 2 pm Eastern Time on the date of publication. For more information, please contact onepress@plos.org.

Kind regards,

Miquel Vall-llosera Camps

Senior Staff Editor

PLOS One

Reviewers' comments:

Reviewer's Responses to Questions

**Comments to the Author**

1. If the authors have adequately addressed your comments raised in a previous round of review and you feel that this manuscript is now acceptable for publication, you may indicate that here to bypass the “Comments to the Author” section, enter your conflict of interest statement in the “Confidential to Editor” section, and submit your "Accept" recommendation.

Reviewer #2: All comments have been addressed

**********

2. Is the manuscript technically sound, and do the data support the conclusions?

The manuscript must describe a technically sound piece of scientific research with data that supports the conclusions. Experiments must have been conducted rigorously, with appropriate controls, replication, and sample sizes. The conclusions must be drawn appropriately based on the data presented.

Reviewer #2: Yes

**********

3. Has the statistical analysis been performed appropriately and rigorously?

Reviewer #2: Yes

**********

4. Have the authors made all data underlying the findings in their manuscript fully available?

The PLOS Data policy requires authors to make all data underlying the findings described in their manuscript fully available without restriction, with rare exception (please refer to the Data Availability Statement in the manuscript PDF file). The data should be provided as part of the manuscript or its supporting information, or deposited to a public repository. For example, in addition to summary statistics, the data points behind means, medians and variance measures should be available. If there are restrictions on publicly sharing data—e.g. participant privacy or use of data from a third party—those must be specified.

Reviewer #2: Yes

**********

5. Is the manuscript presented in an intelligible fashion and written in standard English?

PLOS ONE does not copyedit accepted manuscripts, so the language in submitted articles must be clear, correct, and unambiguous. Any typographical or grammatical errors should be corrected at revision, so please note any specific errors here.

Reviewer #2: Yes

**********

6. Review Comments to the Author

Please use the space provided to explain your answers to the questions above. You may also include additional comments for the author, including concerns about dual publication, research ethics, or publication ethics. (Please upload your review as an attachment if it exceeds 20,000 characters)

Reviewer #2: (No Response)

**********

7. PLOS authors have the option to publish the peer review history of their article (what does this mean?). If published, this will include your full peer review and any attached files.

If you choose “no”, your identity will remain anonymous but your review may still be made public.

**Do you want your identity to be public for this peer review?**  For information about this choice, including consent withdrawal, please see our Privacy Policy.

Reviewer #2: No

**********

# Editor report: Acceptance letter

**DOI:** 10.1371/journal.pone.0354750.r004

**Authors:** Miquel Vall-llosera Camps

PONE-D-26-12553R1

PLOS One

Dear Dr. Kim,

I'm pleased to inform you that your manuscript has been deemed suitable for publication in PLOS One. Congratulations! Your manuscript is now being handed over to our production team.

At this stage, our production department will prepare your paper for publication. This includes ensuring the following:

* All references, tables, and figures are properly cited

* All relevant supporting information is included in the manuscript submission,

* There are no issues that prevent the paper from being properly typeset

You will receive further instructions from the production team, including instructions on how to review your proof when it is ready. Please keep in mind that we are working through a large volume of accepted articles, so please give us a few days to review your paper and let you know the next and final steps.

Lastly, if your institution or institutions have a press office, please let them know about your upcoming paper now to help maximize its impact. If they'll be preparing press materials, please inform our press team within the next 48 hours. Your manuscript will remain under strict press embargo until 2 pm Eastern Time on the date of publication. For more information, please contact onepress@plos.org.

You will receive an invoice from PLOS for your publication fee after your manuscript has reached the completed accept phase. If you receive an email requesting payment before acceptance or for any other service, this may be a phishing scheme. Learn how to identify phishing emails and protect your accounts at https://explore.plos.org/phishing.

If we can help with anything else, please email us at customercare@plos.org.

Thank you for submitting your work to PLOS ONE and supporting open access.

Kind regards,

PLOS ONE Editorial Office Staff

on behalf of

Dr. Miquel Vall-llosera Camps

Staff Editor

PLOS One
